# Women’s empowerment measurements in sub-Saharan Africa: A systematic literature review

**DOI:** 10.1177/17455057251401817

**Published:** 2026-02-16

**Authors:** Immanuel Shipanga, Opeoluwa Oyedele, Lawrence Kazembe

**Affiliations:** 1 Department of Computing, Mathematical and Statistical Sciences, School of Science, University of Namibia, Windhoek, Namibia

**Keywords:** women’s empowerment, sub-Saharan Africa, measurement, decision-making

## Abstract

Quantifying women’s empowerment has gained prominence as a research focus globally. We conducted a systematic review of the literature examining the measurement of women’s empowerment in sub-Saharan Africa (SSA). The objectives of the study is to describe quantitative measurements of women’s empowerment based on individual-level data. We searched PubMed/Medline, Scopus and ScienceDirect databases, along with forward and backward citation tracking, for studies published between 2010 and 2025. The search yielded 1898 records, of which 98 studies met the inclusion criteria. All included studies were peer-reviewed, conducted either across multiple countries or within specific national contexts and analysed data from women of reproductive age. The review revealed considerable variations in the definition of women’s empowerment indicators, the conceptualization of dimensions and a general lack of consensus regarding what specific indicators were intending to measure. This review offers a comprehensive synthesis of the existing quantitative evidence on women’s empowerment measurement in SSA. Furthermore, the findings underscore that empowerment is inherently multidimensional, encompassing aspects such as women’s decision-making, control over resources and autonomy in sexual and reproductive matters. Importantly, the selection of dimensions and indicators is often constrained by the availability of relevant data.

## Introduction

Discrimination that hinders women from attaining good health and achieve fulfilling lives occurs across various levels of society. These constraints are deeply connected to the limited power, influence, choices and autonomy available to women. In addition to promoting gender equality, empowering women is recognized as a key strategy for addressing poverty, hunger and disease, particularly among women in developing countries.

This literature review follows the definition and concept of empowerment by Kabeer^1^ who defined empowerment as a transformative process whereby individuals acquire the ability and power that have been previously denied. This definition views empowerment as a dynamic and interconnected process that includes access to material, human and social resources (pre-conditions), the capacity to set and pursue personal goals (agency) and the realization of desired outcomes (achievement).^2^ Recently, more than ever before, scholarly attention has turned to the subject of women’s empowerment. Proponents of women’s empowerment have highlighted its importance, not only as a human right but also to attain health and population goals.^3^ Empirically, there is a link between the Women’s empowerment and associated health outcomes, as well as lower fertility preference,^4^ family planning, children’s health and nutrition,^5–8^ improvements in maternal health^6,9^ contraceptive use^10^ and use of antenatal care.^11–14^ Moreover, the quest to meaningfully measure women’s empowerment is becoming increasingly important in sub-Saharan Africa (SSA) where development is lagging.

As women’s empowerment gains prominence in research across various regions, a wide range of measurement approaches has become increasingly common in the global development agenda. While such measures are important, scholars have struggled to agree on a universal metric to measure women’s empowerment. The adversity faced by scholars may be due to the different forms that empowerment can take, depending on the conditions relating to the individual and their environment. To enhance understanding of the challenges in measuring women’s empowerment, we conducted a systematic literature review examining how women’s empowerment has been measured in SSA and further aimed at summarizing the indicators based on individual-level data and methodologies used to derive them.

### Theoretical framework

This study is grounded in liberal feminist theory, which advocates for comprehensive gender equality and the empowerment of women by promoting their access to public institutions and prioritizing women’s issues in national discourse.^15^ Liberal feminism emphasizes the importance of educational reforms and the implementation of appropriate legislation as key strategies to address gender disparities. These measures aim not only to reduce inequality between men and women but also to transform societal norms and beliefs.^16^

The initial measures of gender dimension of development were done in the 1990s via the United Nations Development Programme (UNDP)’ gender related development index and gender empowerment measure drawing on Amartya Sen’s capability approach.^17^ Since then, various indices have been developed by different researchers using different methodologies to measure gender inequality and women’s empowerment.

Together with criticism of the UNDP global indices, the complexity of measuring empowerment emerged. According to Ewerling et al.^17^ and Asaolu et al.,^18^ empowerment is understood to possess several key characteristics: (i) it is a latent construct, meaning that observed behaviours serve only as proxies for an underlying phenomenon; (ii) it can be either intrinsic, reflecting internal agency or instrumental, serving as a means to an end; (iii) it may be universal or context-specific, varying across cultural and social settings; (iv) it can be achieved individually or collectively, indicating that empowerment operates at multiple levels, including the individual, household, community and national levels; (v) it spans various dimensions such as familial, economic, psychological, socio-cultural, legal and political each offering a different lens of application; and (vi) its assessment depends on what is being measured, who is doing the measuring (self-assessment or external evaluation) and the methodology employed, whether qualitative or quantitative.

Numerous indicators were used to capture the complex and multidimensional construct, encompassing various indicators, along with various conditions and factors that influence it. Kishor and Gupta^19^ argued that in addition to the indicators of evidence of empowerment, indicators pertaining to access to potential sources for empowerment as well as indicators for setting for empowerment is crucial in operationalizing women’s empowerment. Many of these indicators, particularly those related to women’s empowerment, have been incorporated into nationally representative Demographic and Health Surveys (DHSs). Key indicators of women’s status and empowerment commonly used in national surveys include: literacy and educational attainment; employment status and type of occupation; control over personal earnings; age at first marriage and first birth; contraceptive use; spousal age and education differences; participation in household decision-making; attitudes towards wife-beating; beliefs about a woman’s right to refuse sex with her husband; and barriers women face in accessing healthcare for themselves. In certain countries, additional questions were included to empowerment measures such as autonomy in spouse selection, support from the natal family, ownership of assets, financial decision-making for different purposes, awareness and participation in microcredit initiatives, attitudes towards gender roles, freedom of movement, membership in formal or informal associations and access to banking services.

## Materials and methods

### Search strategy

We conducted an electronic database search for articles on women’s empowerment on 10 May 2023, with an update completed on 10 June 2025. The databases included PubMed, Scopus and ScienceDirect. The following search terms were used individually and in various combinations: ‘female empowerment’, ‘women’s empowerment’, ‘women empowerment’, ‘women’s autonomy’ and ‘sub-Saharan Africa’ to identify research studies published between 2010 and 2025. The search strategy also incorporated a manual search to identify additional relevant research on the same topic.

### Inclusion criteria

In this systematic literature review, studies were considered eligible if they were (i) published in the English language between 2010 and 2025; (ii) applied quantitative analysis; (iii) examined female/women’s empowerment either as an independent variable or dependent variable and described how it was measured; (iv) conducted or analysed data from countries in the SSA region and (v) that relied upon individual level data. The research titles and abstracts were evaluated against these inclusion criteria.

All studies that met the inclusion criteria were imported to Rayyan (a web application designed to facilitate the screening process for researchers working on systematic reviews, scoping reviews and other literature review projects) and duplicates were removed followed by the manual revision. At the first stage of study selection, titles and abstracts of the remaining articles were independently screened by two reviewers (IS and OO). To ensure the quality of the assessment, each study was critically appraised using the Critical Appraisal Skills Programme checklist (Supplemental Table S2). The appraisal focused on key aspects including the validity of the study design, methodology and reported results. Based on these criteria, each study was rated as having a high, moderate or low risk of bias. In the event of conflicts over inclusion, a third reviewer (LK) was involved for resolution. The full texts of the articles that satisfied the inclusion criteria were obtained and examined, while articles that did not fit the inclusion criteria were excluded. Articles with uncertain eligibility were retained for full-text review. The articles selected in the previous screening phase were reviewed in full, and relevant data were manually extracted and independently recorded in a structured spreadsheet by each of the three reviewers. The key information extracted from each article included authorship, title, study location, data, participant details, women’s empowerment and measures of women’s empowerment. Moreover, reference lists of the included studies were screened for further relevant documents. Any disagreements regarding article inclusion were resolved through consensus. All analyses were based on previously published articles; therefore, no ethical approval or patient consent was required.

## Results

### Search results

**Figure 1.**
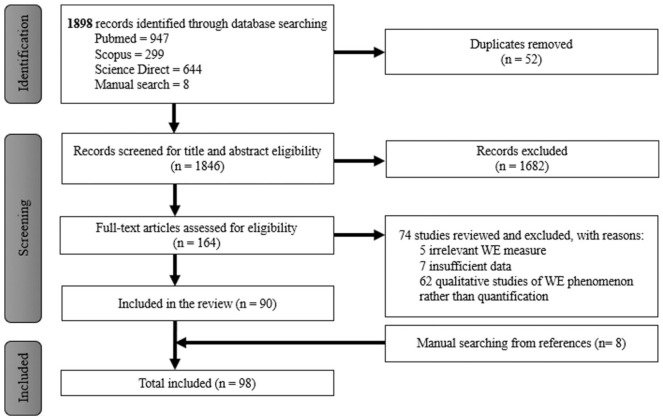
Flowchart of the study selection procedure.

The search yielded a total of 1898 publications across all databases used. After removing duplicates, 1846 articles were screened based on title and abstract, of which 1682 were excluded. A total of 164 full-text articles were then assessed for eligibility, leading to the further exclusion of 74 articles. These 74 articles were excluded with reasons such as (i) irrelevant women’s empowerment (WE) measure due to the usage of non-WE oriented indexes, (ii) insufficient data due to the lack of explanation on how WE was measured in their study and (iii) qualitative studies of WE phenomenon rather than quantifying it. This resulted in 90 publications being included in the review. Additionally, the manual searching identified eight more articles. Therefore, the final number of publications included in this review was 98, as shown in Figure 1. Supplemental Table S3 shows the characteristics of the 98 studies included in this review. Of the 98 publications included, 95.9% (*n* = 94) were rated as having a low risk, while 4.1% (*n* = 4) were rated as having a moderate risk (Supplemental Table S2).

### Study characteristics

All selected publications were published as peer-reviewed papers and proposed women’s empowerment measures based on data collected at the individual level measures. Of the 99 publications, 78.6% (*n* = 77) relied on data exclusively from the nationally representative DHSs, 3.1% (*n* = 3) used DHSs data with the Indian National Family Health Survey,^20^ DHS together with Multiple Indicator Cluster Survey (MICS)^21,22^ and the remaining 18.4% (*n* = 17) were based on other surveys or primary data sources. In addition to studies conducted in SSA countries, the analyses also included five publications^20,23–26^ that drew on data from both SSA and other low-and middle-income countries. Approximately 55.1% (*n* = 54) of the publications were based on data from individual countries within the region namely: Uganda,^10,27–29^ Nigeria,^6,30–35^ Ethiopia,^13,36–45^ Benin,^46^ Zambia,^47^ Kenya,^48–51^ Democratic Republic of Congo (DRC),^52,53^ Burkina Faso,^54,55^ Namibia,^56^ Zimbabwe,^57,58^ Sierra Leon,^42,59^ Ghana,^60–65^ Mozambique,^66,67^ Cameroon,^14,68^ Tanzania,^69,70^ Rwanda,^71,72^ Chad^22^ and the Gambia.^73^ The remaining 44 studies^4,7–9,11,12,18,20,21,23–26,33,74–103^ utilized pooled data from multiple SSA countries, allowing for comparative analyses across countries within the region and, in some cases, beyond.

Given the specific survey design, most DHS-based publications analysed the empowerment of women of reproductive age (15–49 years). However, two studies diverged from this: one focused on women aged 35 years and above,^4^ and another on women aged 20–49 years.^70^ Four studies specifically targeted rural-based married or partnered women,^34,44,60,90^ while one study centred on urban-based women.^31^ Seven studies^43,52,62,86,89,97,99^ examined women with children under the age of 5 and two studies analysed responses from couples.^9,48^ One study^23^ utilized data from the World Bank Annual Report and applied global indices developed by the United Nations, that is, the gender gap index, gender equity index and social institutions and gender index. As a result, the study did not involve a specific population sample. The characteristics of the publications and the corresponding reference are presented in Tables 1 and 2.

**Table 1.** Characteristics of the publications proposing a women’s empowerment measure (*n* = 98).

Characteristics	DHS-based publications	DHS and other surveys	Other surveys incl. primary data-based publications
Publication type	• Peer-reviewed papers^7–12,14,18,25–27,29,32,33,35,36,40,41,43,45,49,50,52,56–60,62–64,67–80,82–94,96–106^	• Peer-reviewed papers^20–22^	• Peer-reviewed papers^6,13,23,30,31,34,37,39,42,44,46–48,51,53,54,65,95^
Study location	• Multiple countries (two or more) from different • region^24–26^ • Multiple sub-Saharan publications^4,7–9,11,12,18,33,74–94,96–103^ • Sub-Saharan country specific publications ○ Uganda^27–29^ ○ Nigeria^32,33,35^ ○ Ethiopia^36,38,40,41,43^ ○ Kenya^49,50^ ○ DRC^52^ ○ Burkina Faso^55^ ○ Namibia^56^ ○ Zimbabwe^57,58^ ○ Sierra Leon^59,105^ ○ Ghana^10,60–64^ ○ Mozambique^66,67^ ○ Cameroon^14,68^ ○ Tanzania^69,70^ ○ Rwanda^71,72^ ○ Chad^22^ ○ Gambia^73^	• Multiple countries (two or more) from different regions^20^ • Multiple sub-Saharan studies^21^ • Sub-Saharan country specific studies ○ Chad^22^	• Multiple countries (two or more) from different regions^23^ • Multiple sub-Saharan studies^95^ • Sub-Saharan country specific publications ○ Nigeria^6,30,31,34^ ○ Ethiopia^13,37,39,42,44,45^ ○ Benin^46^ ○ Zambia^47^ ○ Kenya^48,51^ ○ DRC^53^ ○ Burkina Faso^54^ ○ Ghana^65^
Population	• Married/partnered women aged 15–49 years^7,8,12,18,24,25,27,31,36,38,45,49,50,55,61,63,64,69,72–77,80,88,92,93,96,98,100,104–106^ • Women 15–49 years^10,14,26,28,29,32,33,35,40,41,56,58,59,66,67,71,78,79,81,85,87,91,94,101–103^ • Women 15–49 with children <5 years^43,52,62,86,89,98,99^ • Women 20–49 years^70^ • Women 35 years and above^49^ • Rural-based married/partnered women 15–49 years^60,90^ • Married/partnered women aged 15–49 and men aged 15–59 years^9,48^	• Married/partnered women aged 15–49 years^20–22^	• Married/partnered women aged 15–49 years^6,34,39,42,44,95^ • Women 15–49 years^13,30,37,46,51,53,65^ • Women 15–49 years with children <5 years^47,48,54^ • Rural-based women 15–49 years^34,44^ • Urban-based women aged 15–49^31^

DHS: Demographic and Health Survey; DRC: Democratic Republic of Congo.

**Table 2.** Measures of women’s empowerment in SSA, indicators and publications.

Dimensions/domains	Sub-dimension/sub-domain	Women’s empowerment indicators and publications
Familial/interpersonal empowerment	Decision-making or women autonomy (*n* = 74)	Person who decides on respondents’ healthcare, on large household purchases, daily household purchases and on visit to family or relatives^9,13,30,74,102^; person who decides on respondent’s own income, husband income spending, respondent’s healthcare, household earnings and household purchases and visiting family^27,50,52,71,79,80,82,85,92^; person who decides on respondent’s healthcare, on large household purchases, on visit to family or relative^8,12,14,22,31,32,34,36,44,55,59,63,66,67,70,72,78,88,93,94,104^; decision-maker on respondents use of contraceptives, responder’s healthcare, large household purchases, daily household purchases and visit to family^20^; decision-maker on respondent’s healthcare, major household purchases, spending respondent money and visiting relatives^7,10,28,29,51^; decision-maker on members of household’s healthcare, regarding decisions on kids’ schooling and regarding whether to have another child or not^46^; decision-making power^37^; person who decide on respondent’s healthcare, large household purchases, schooling children and healthcare of children^47^; decision-maker on large household purchases, purchase of daily household needs, visiting female partner’s family or relatives, spending the money female partner earns and own heathcare/how many children to have^48^; person who decides on respondent’s healthcare, on larger household purchases, on visit to family or relatives and on household finances^38^; decision-maker on spending of household income^81^; decision-maker on use a contraceptive method and have another child, she to consult a doctor, when she was pregnant and what to do when a child was sick, she contributed to use a contraceptive method and to decision to have another child^54^; decision-maker on respondent’s healthcare, large household purchases, money husband earn and visiting family or relatives^24,40,69,84,86^; decision-maker on respondent’s healthcare and purchase of household goods^21,41,43,45,64,89,91,97–101,103^; decision-maker on respondent’s earning, on husbands earning, on household purchases, decision on visiting family, healthcare, contraceptive use, personal of family health and on using a condom if husband has STD^57^; decision-maker on visiting family/relatives, healthcare, household purchases and household earning^83^; permission to get medical help, getting money for treatment, wanting to go alone to health centres, on respondent healthcare, on large household purchases, on visit to family or relatives on what to do with husband earnings^11^; decision-maker on respondent’s healthcare, house earning, household purchases and visiting family members^60,61,87,105^; decision-maker on large household purchases, own health, going to public meetings^42^; decision-maker on large household purchases, small household purchases, visit family, deciding when and where to seek medical health^6^; decision-maker on expenditure of woman earning, partners income, large household purchases, healthcare and visit relatives^68^; person who decides on large household purchases, contraceptive use and self-earnings^73^; decision-maker on large household purchases, daily purchase, medical purchases, clothes purchases^95^; decision-maker on healthcare and purchase of household goods^58,96^; decision-maker on large household purchases, daily purchases, healthcare, buying clothes and how partners earning will be used^53^; decision-maker on daily purchases, large household purchases, can refuse sex, permission to health facility and visit family and friends^65^; decision-maker on woman’s healthcare, significant household purchases, daily household needs, visits to family or relatives, daily food choices and allocation of her partner’s earnings^26^.
	Financial autonomy (*n* = 2)	Respondents earn income, own saving, spending cash earnings and have influence on husband income^39^; respondent have savings, access to financial information and financial goals.^95^
	Familial/interpersonal (*n* = 2)	Respondent’s control over domestic decision-making, control over sexual relations, marriage, fertility, contraception, health-seeking behaviour and attitudes regarding domestic violence^75^; decision-maker on healthcare, major household purchases, daily household needs, visiting relatives, money husband earns, refuse to have sex-husband has STD, have other women, if tired/not in the mood, on use of condom if husband has STD, decision-maker on contraceptive use, husband knows she is on contraceptives.^56^
	Socio-familial (*n* = 7)	Freedom of mobility, control of own health, free of domestic violence^76^; decision-making power^46^; decision-making regarding family visits, women’s own health and attitude towards domestic violence^62^; attitude towards violence, household decision-making, life course indicator, land and home ownership^106^; household decision-making, attitude towards violence, age of cohabitation and first birth^33^; earning gap.^26^
	Partner prohibition (*n* = 2)	Respondent prohibited from working outside home, visit from people, visit friends, visit family, using phone^6^; prohibited from using contraceptives.^30^
	Access to healthcare (*n* = 12)	Women’s ability to refuse sex or ask a partner to use condoms^4,18,33,106^; getting help permission for medical help, money for treatment, distance to a health facility, not wanting to go alone for medical help^11,24,40,55,69^; access to healthcare^90^; distance to family, getting medical help^70^; sexual and reproductive health, control over healthcare.^25^
	Mobility (*n* = 4)	Respondent requires permission to go to the market, to health centre and to visit friends^46^; Decision to visit family or relatives, healthcare and social affairs^39^; Partner influence^53^; Freedom of movement.^13^
	Family (*n* = 1)	Age at first birth, age at first sex, decision on household purchases, decision on contraceptive use, visiting family, respondents’ healthcare, heard of family planning on radio, heard of family planning on TV, read family planning on newspaper.^4^
	Material environment (*n* = 1)	Control over household purchases, control over earning.^25^
	Social relationships (*n* = 1)	Autonomy in social relationships, freedom of association.^25^
Socio-cultural/psychological empowerment	Attitudes towards wife beating/justifying violence (*n* = 76)	Beating justified if wife goes out, neglect children, argues with him, burns food^9,27^; who decided whether they could visit their family and relatives^75^; beating justified if wife goes out, neglects children, argues with him, refuses sex, burn food, suspected of unfaithful, refuses to have another child^6,30^; beating was justified if the wife went out without permission of husband, neglected the children, argued with husband, refused sex with husband or burned the food^7,8,11,12,14,20,24,40,42,55–57,60,61,66,67,69,73,78,79,82,83,85,87,92,94,102,105^; Beating justified if wife goes out, neglect children, argues, refuses sex, burn food, had sex outside marriage, wife justified refusing sex if husband have sex with other women and to ask for condom if husband has STD^50,80^; equal effectiveness of boys and girls at school, reports on female genital mutilation, changing socio-cultural things in the community^37^; attitude towards violence, household decision-making, life course indicator, land and home ownership^18,38,106^; beating justified if wife neglects children, refuse to have sex, burn food^21,41,43,45,64,72,81,89,91,96–101,103^; beating justified if wife goes out without telling husband, neglect children, refuse sex^84^; decision-making regarding family visits, women’s own health and attitude towards domestic violence under five scenarios^62^; household decision-making, attitude towards violence, age of cohabitation and first birth^33^; women’s perception and experience of violence and abuse^86^; women attitude towards violence^90^; beating justified if wife goes out without telling husband, if she neglects the children, if she burn the food and if she argues with the husband^70^; agreement on beating justified – by any reason or excuse, to have sex with partner, ever been through unwanted – sexual intercourse with partner, nonintercourse acts by partner.^58^
	Socio-cultural or self-esteem (*n* = 5)	Who decided whether they could visit their family and relatives^75^; self-esteem indicators^37^; education, frequency of listening to radio, reading a newspaper^4^; attitude towards violence, household decision-making, life course indicator, land and home ownership^49^; locus of control, self-esteem.^13^
	Attitudes towards refusing sexual intercourse/sexual empowerment (*n* = 9)	Respondent can refuse sex, when husband has STD, has other women, has recently given birth, tired or not in mood^9^; if husband has STD, has intercourse with other women, tired not in mood^20^; women’s ability to refuse sex or ask a partner to use condoms^11,29,66,67,71,78^; control over sexuality.^53^
	Physical integrity (*n* = 1)	Acceptance of physical violence and intimate partner violence.^25^
	Free from spousal violence (*n* = 1)	Ever physical, emotional, economic and sexual violence.^39^
	Influencer (*n* = 4)	Attitudes towards wife beating.^34,35,59,93^
	Leadership (*n* = 1)	Sense of self and confidence.^46^
Economic Empowerment	Economic or economic freedom (*n* = 20)	Income contribution relative to husband, decision on how each partners income would be used and decision about major and daily household purchases^75^; ownership to property^27^; income generation, control over finance^76^; access to money of her own^30^; income generation, control over finance^77^; work status, relationship household head, control over earning and land ownership^10^; afford fruits and vegetable, own clothes own beauty purchase^46^; income generating by women, asset ownership^37^; working, type of earning, own a house, own land^4,57^; respondent employed, deciding on spending money, amount of earning^56^; women worked in the last 12 months, who does woman work for, the type of earnings from woman’s work, type of occupation, work all years^11^; control over women’s income, decision-making on large household purchases, women’s ability to work outside home^62^; work/labour force participation, legal status^33^; respondent worked, own land and own house^68^; women’s owning of a house, land and the type of earning from her work^29,71^; participation in income activity^86,90^; cash earnings.^44^
	Labour force participation (*n* = 18)	Occupation, type of earning, seasonality of occupation and income ratio^4,18,106^; respondents currently working.^13,14,24,26,28,51,60,73,79,82,83,94,102,105^
	Access to or control over resources/resources (*n* = 7)	Ownership of major durable items, land and house^39^; seasonality, income relative to partner^84^; land ownership, ownership of productive assets, having independent source of income and making household purchase using their income^51^; occupation, having a mobile phone etc.^34,35,59,93^
	Property ownership (*n* = 7)	Respondent own land and respondent own a house.^11,40,44,61,69,70,92^
Human and social assets	Human and social assets (*n* = 5)	Schooling attainment, age at first sex, age at first cohabitation, age at first birth, spousal age difference, spousal schooling difference, spousal earning difference, first sex at marriage, work for cash or in-kind^41,80^; age at first sex, age at first cohabitation, age at first birth^7^; women’s education level, women’s occupation and age at first marriage.^72,73^
	Family (*n* = 1)	Age at first birth, age at first sex, decision on household purchases, decision on contraceptive use, decision to visiting family, decision on respondents’ healthcare, heard of family planning on radio, heard about family planning on TV, read about family planning from newspaper.^4^
	Education/social independence/socio-economic status (*n* = 26)	Educational attainment^31,44,51,73,90^; literacy, highest education level and spousal difference in educational attainment^18,33,49,106^; highest education, ability to read a sentence^57^; worked in last 12 months, age at cohabitation, age at first birth and age difference between wife and husband^21,41,43,45,64,89,91,96–101,103^; highest education level, frequency of reading newspaper, frequency of watching TV, frequency of internet use, respondent has a bank account, own a mobile phone, employment, own a phone, frequency of watching TV, frequency of reading newspaper, type of earning and educational attainment^69^; member in community groups.^44^
	Knowledge level or exposure to media or awareness (*n* = 14)	Listening to radio, reading newspaper/magazine, watching TV and educational level^60,79,82,83,85,87,94,102,105^; unmet need, heard family planning on radio, heard family planning on TV, read about family planning on print media or text message, knowledge about contraceptive methods^11^; watching TV, reading newspaper or magazine, listening to the radio, heard about family planning on the radio, heard family planning on TV, from newspapers^24^; women’s ability to have the opportunity to read a newspaper or a magazine, listen to the radio and watch TV.^29,71^
	Age at critical events/reproductive self-determinations (*n* = 2)	Age of the respondent at first cohabitation and age at first birth.^58,69^
Others	Legal (*n* = 5)	Legal rights over land and houses^76,77,86^; knowledge of women legal rights^37^; women’s judicial and legislative entitlements over land and over house ownership.^62^
	Political/non-family group membership (*n* = 2)	Member of a non-family group^46^; political representation.^37^
	Male involvement (*n* = 1)	Help from adult male.^46^
	Spousal communications (*n* = 1)	Domestic activities, expenses, child health, nutrition, personal health.^54^
	United Nation global indices (*n* = 1)	Gender gap index, GEI and SIGI.^23^

GEI: gender equity index; SIGI: social institutions and gender index; SSA: sub-Saharan Africa; STD: sexual transmited diseases.

### Women’s empowerment measures

Measuring women’s empowerment is inherently complex. This is reflected in the fact that all publications consistently acknowledged its multidimensional nature, highlighting that empowerment encompasses various overlapping and context-specific domains. The review revealed varying terminology used to describe its components of empowerment. Terms such as empowerment areas, components, dimensions, domains, indicators, indices, measures and variables were used interchangeably. Notably, there is no established consensus on the hierarchical structure or comparability of these terms across selected studies.

In Table 2, we listed dimension/domains along with the corresponding variables, as assigned by the study authors. For ease of presentation, the various sub-dimensions were grouped into four overarching dimensions: familial/interpersonal, socio-cultural/psychological, economic and human and social assets empowerments. An additional category, termed others, was used for sub-dimensions that did not fit neatly into the four overarching dimensions. The average number of dimensions used to measure women’s empowerment was 3.3, ranging from a single dimension, primarily based on DHS-derived decision-making/women autonomy indicators^22,32,38,47,52,63,65,74,104^ to 11 dimensions in studies utilizing primary data.^13^

The most commonly used dimension of women’s empowerment, identified in 76 studies, was socio-cultural/psychological empowerment. This dimension was commonly measured through a set of questions assessing whether a woman believes a husband or partner is justified in beating his wife under specific circumstances. These scenarios typically included: going out without the husband’s permission, neglecting the children, arguing with the husband, refusing sex or burning the food. This construct, referred to variously as attitudes towards violence, perceptions of violence, violence against women or attitudes towards wife-beating, were used in 28 studies,^7,8,10–12,14,20,24,40,42,55–57,60,61,66,69,73,78,79,82,83,85,87,92,94,102,105^ making it the most consistently applied set of indicators for this dimension.

The familial/interpersonal empowerment, focusing on women’s decision-making power or women’s autonomy was the second most reported subcategory of women’s empowerment used in 74 publications. While certain studies have categorized dimensions such as financial autonomy,^39,95^ partner prohibition^6,30^ and access to healthcare,^11,18,24,40,49,55,69,106^ etc. the variables within these domains predominantly capture women’s decision-making capacities and their dependence on partners for issues pertaining to their own well-being and that of household members.

Most publications (*n* = 25) in this dimension combined two or three variables of decision-making mostly found in the DHS questionnaire such as the person responsible – respondent’s healthcare, on large household purchases, on visit to family or relatives.

Other notable categories included education/social independence, addressed in 26 studies and economic empowerment and labour force participation, which were collectively featured in 28 publications. Additionally, knowledge level was examined in 14 studies, while access to healthcare indicators appeared in 12 studies. All remaining sub-dimensions and subdomains were covered in fewer than 10 publications. Rather than developing their own measures, several studies employed established frameworks such as the African-based Survey-Based Women’s Empowerment Index (SWPER),^21,41,43,64,89,91,97–99,101,103^ the updated version SWPER global^45,100^ and the Harvard Gender Roles Framework.^35,59,93^

## Discussion

This study provided a comprehensive appraisal of evidence describing women’s empowerment measures in SSA. The findings from this study help to paint a complete picture of the complex women’s empowerment measurement in relation to gender and development monitoring in the region. The literature review revealed a great heterogeneity of definitions and dimensions of women’s empowerment which constitute a challenge in defining a measure suitable for comparison across population and over time.

Despite satisfying interest in the subject, many studies involved DHS data which indicate the importance of the survey to women’s empowerment measurement construct. The results revealed that the majority of DHS-based studies used data collected from mostly East and West African countries. Although some studies that focused on MICS data included Southern African countries, country-specific studies from within the region were limited. Identified studies were conducted in the DRC,^52,53^ Mozambique,^66,67^ Namibia,^56^ Tanzania,^69^ Zambia^47^ and Zimbabwe.^57,58^ The relatively low number of studies from Southern Africa may be attributed to the prioritization of other regions (east and west Africa) by international and regional development agencies, often based on perceived developmental needs, larger population sizes, higher fertility rates and greater health burdens.

The DHS datasets play a pivotal role in providing a standardized measurement which are beneficial for comparing various populations in diverse cultures. However, context-specific measures can provide opportunities to reflect women’s life experiences in the contexts in which they live.^105^ Such studies, in particular the ones that applied primary data provided additional indicators to the measure which is not available in the DHS standard questionnaire. Moreover, the degree of women’s empowerment is affected by numerous elements at various intrapersonal, interpersonal and ecological levels. Hence, social, economic and cultural systems are (very) crucial in defining the boundaries of empowerment in specific contexts. Other factors, such as the availability and accessibility of health services, women’s position in society and the cultural expectations of women, effectively influence women’s empowerment regardless of their individual or household characteristics.

Household decision-making, attitudes towards violence and control over sexual relations were the most frequent measures of women’s empowerment used in the reviewed studies of this systematic review. This is not surprising, as women’s decision-making was one of the earliest ways of operationalizing women’s empowerment, on which the DHS – commonly used dataset for women’s empowerment measures – is based. The frequent use of decision-making and attitudes towards violence as core domains of women’s empowerment in SSA is contextually appropriate, given the prevailing cultural norms and patriarchal systems that have historically excluded women from decision-making processes and often justified violence as a means of enforcing culturally prescribed gender roles and behaviours.

This systematic review study had several potential limitations. Firstly, most of the research studies reviewed in this study used data from the DHS, which exclude never-married women as a focus of their study. Secondly, only a few studies included matched couples or male perspectives on women’s empowerment and fertility preferences. Collecting information from both partners and analysing it can provide the opportunity to account for men’s characteristics and opinions on women’s empowerment.

## Conclusion

This systematic review provided a comprehensive synthesis of the available quantitative evidence of women’s empowerment measurement in SSA. It revealed considerable variations in the definition of women’s empowerment indicators, the conceptualization of dimensions and a general lack of consensus regarding what specific indicators were intending to measure. The most frequently employed dimensions in the measurement of women’s empowerment included decision-making authority, attitudes towards gender-based violence and control over sexual relations. Moreover, this review confirms that measuring empowerment is multidimensional, spanning women’s decision-making and control over resources and sexuality. It is noteworthy that qualitative research is essential for defining the various dimensions of women’s empowerment and outlining the ways in which women may be more empowered. However, the choice of dimensions to include was limited by the availability of data. More studies on women’s empowerment in the Southern African region of the SSA are further recommended to be carried out. In addition, context-specific studies with a specific focus on Southern Africa are required to add to the few existing literatures of women’s empowerment measure using primary data.

## Supplemental Material

sj-docx-1-whe-10.1177_17455057251401817 – Supplemental material for Women’s empowerment measurements in sub-Saharan Africa: A systematic literature review Supplemental material, sj-docx-1-whe-10.1177_17455057251401817 for Women’s empowerment measurements in sub-Saharan Africa: A systematic literature review by Immanuel Shipanga, Opeoluwa Oyedele and Lawrence Kazembe in Women's Health
