# Supplementary material for: Women’s empowerment measurements in sub-Saharan Africa: A systematic literature review
Source: Womens Health (Lond). 2026 Feb 16;22:17455057251401817. doi: 10.1177/17455057251401817 (PMC12924980; doi:10.1177/17455057251401817)
Supplement: sj-docx-1-whe-10.1177_17455057251401817 – Supplemental material for Women’s empowerment measurements in sub-Saharan Africa: A systematic literature review [file sj-docx-1-whe-10.1177_17455057251401817.docx]

Table S1: Search Strategy

| Search query | Database | Results |
| --- | --- | --- |
| ((((Women's[All Fields] OR ("female"[MeSH Terms] OR "female"[All Fields])) AND ("empowerment"[MeSH Terms] OR "empowerment"[All Fields])) AND "autonomy"[All Fields]) AND agency[All Fields]) AND ("africa south of the sahara"[MeSH Terms] OR ("africa"[All Fields] AND "south"[All Fields] AND "sahara"[All Fields]) OR "africa south of the sahara"[All Fields] OR ("sub"[All Fields] AND "saharan"[All Fields] AND "africa"[All Fields]) OR "sub saharan africa"[All Fields]) | Pubmed  10 June 2025 | 947 |
| women&apos;s OR female AND  empowerment AND autonomy AND sub-saharan AND africa AND ( LIMIT-TO ( SUBJAREA , "ARTS" ) ) | Scopus  10 June 2025 | 299 |
| women empowerment AND Sub-saharan Africa | Science Direct  10 June 2025 | 644 |
| None | Manual search  10 June 2025 | 8 |
| **Total number of studies identified** |  | **1898** |

Table S2: Critical Appraisal Skills Programme (CASP) checklist

| **Author** | **Year** | **Title** | **D1** | **D2** | **D3** | **D4** | **D5** | **D6** | **D7** | **D8** | **D9** | **D10** | **D11** | **Overall** |
| --- | --- | --- | --- | --- | --- | --- | --- | --- | --- | --- | --- | --- | --- | --- |
| Hindin and Muntifering | 2011 | Women's autonomy and timing of most recent sexual intercourse in sub-Saharan Africa: A multi-country analysis | Low | Low | Low | Low | Low | Low | Low | Low | Low | Low | Low | Low |
| Upadhyay and Karasek | 2012 | Women’s Empowerment and Ideal Family Size: An Examination of Empowerment Measures in Sun-Saharan Africa | Low | Low | Low | Low | Low | Low | Low | Low | Low | Low | Low | Low |
| Do and Kurimoto | 2012 | Women's empowerment and choice of contraceptive methods in selected African countries. | Low | Low | Low | Low | Low | Low | Low | Low | Low | Low | Low | Low |
| Kwagala et al. | 2013 | Empowerment, partner's behaviours and intimate partner physical violence among married women in Uganda. | Low | Low | Low | Low | Low | Low | Low | Low | Low | Low | Low | Low |
| Jennings et al. | 2014 | Women's empowerment and male involvement in antenatal care: analyses of Demographic and Health Surveys (DHS) in selected African countries. | Low | Low | Low | Low | Low | Low | Low | Low | Low | Low | Low | Low |
| Corroon et al. | 2014 | The role of gender empowerment on reproductive health outcomes in urban Nigeria. | Low | Low | Low | Low | Low | Low | Low | Low | Low | Low | Low | Low |
| Solanke | 2015 | Marriage Age, Fertility Behaviour, and Women's Empowerment in Nigeria | Low | Low | Low | Low | Low | Low | Low | Low | Low | Low | Low | Low |
| Na et al. | 2015 | Association between women's empowerment and infant and child feeding practices in sub-Saharan Africa: an analysis of Demographic and Health Surveys. | Low | Low | Low | Low | Low | Low | Low | Low | Low | Low | Low | Low |
| Shimamoto and Gipson | 2015 | The relationship of women's status and empowerment with skilled birth attendant use in Senegal and Tanzania. | Low | Low | Low | Low | Low | Low | Low | Low | Low | Low | Low | Low |
| Ibrahim et al. | 2015 | The Influences of Women's Empowerment on Reproductive Health outcomes: A comparative study of Nigeria and Uttar Pradesh State (India). | Low | Low | Low | Low | Low | Low | Low | Low | Low | Low | Low | Low |
| Asaolu et al. | 2016 | Association between Measures of Women’s Empowerment and Use of Modern Contraceptives: An Analysis of Nigeria’s Demographic and Health Surveys. | Low | Low | Low | Low | Low | Low | Low | Low | Low | Low | Low | Low |
| Blackstone | 2017 | Women’s Empowerment, Household Status and Contraception Use in Ghana. | Low | Low | Low | Low | Low | Low | Low | Low | Low | Low | Low | Low |
| Lan and Tavrow | 2017 | Composite measures of women's empowerment and their association with maternal mortality in low-income countries. | Low | Low | Low | Low | Low | Low | Low | Low | Low | Low | Low | Low |
| Tiruneh et al. | 2017 | Women's autonomy and maternal healthcare service utilization in Ethiopia. | Low | Low | Low | Low | Low | Low | Low | Low | Low | Low | Low | Low |
| Alaofe et al. | 2017 | Association Between Women's Empowerment and Maternal and Child Nutrition in Kalale District of Northern Benin. | Low | Low | Low | Low | Low | Low | Low | Low | Low | Low | Low | Low |
| Bayissa et al. | 2017 | The Multidimensional Nature of Women’s Empowerment: Beyond the Economic Approach | Low | Low | Low | Low | Low | Low | Low | Low | Low | Low | Low | Low |
| Asaolu et al. | 2018 | Measuring Women’s Empowerment in Sub-Saharan Africa: Exploratory and Confirmatory Factor Analyses of the Demographic and Health Surveys. | Low | Low | Low | Low | Low | Low | Low | Low | Low | Low | Low | Low |
| Yaya et al. | 2018 | Women’s Empowerment as an Enabling Factor of Contraceptive Use in Sub-Saharan Africa: A Multilevel Analysis of Cross-sectional Surveys of 32 Countries. | Low | Low | Low | Low | Low | Low | Low | Low | Low | Low | Low | Low |
| Ford et al. | 2018 | Women's decision-making and uptake of services to prevent mother-to-child HIV transmission in Zambia. | Low | Low | Low | Low | Low | Low | Low | Low | Low | Low | Low | Low |
| Voronca et al. | 2018 | Relationship between empowerment and wealth: trends and predictors in Kenya between 2003 and 2008-2009. | Low | Low | Low | Low | Low | Low | Low | Low | Low | Low | Low | Low |
| Miedema et al. | 2018 | Women’s empowerment in East Africa: Development of a Cross-country Comparable Measure. | Low | Low | Low | Low | Low | Low | Low | Low | Low | Low | Low | Low |
| Ebrahim and Atteraya | 2019 | Women's Household Decision-Making and Intimate Partner Violence in Ethiopia | Low | Low | Low | Low | Low | Low | Low | Low | Low | Low | Low | Low |
| Atake and Gnakou-Ali | 2019 | Women’s Empowerment and Fertility Preferences in High Fertility Countries in Sub-Saharan Africa. | Low | Low | Low | Low | Low | Low | Low | Low | Low | Low | Low | Low |
| Mengo et al. | 2019 | Marital Rape and HIV Risk in Uganda: The Impact of Women's Empowerment Factors. | Low | Low | Low | Low | Low | Low | Low | Low | Low | Low | Low | Low |
| Chol et al. | 2019 | Women's autonomy and utilisation of maternal healthcare services in 31 Sub-Saharan African countries: results from the demographic and health surveys, 2010-2016. | Low | Low | Low | Low | Low | Low | Low | Low | Low | Low | Low | Low |
| McKenna et al. | 2019 | Women's decision-making power and undernutrition in their children under age five in the Democratic Republic of the Congo: A cross-sectional study. | Low | Low | Low | Low | Low | Low | Low | Low | Low | Low | Low | Low |
| Heckert et al. | 2019 | Is women's empowerment a pathway to improving child nutrition outcomes in a nutrition-sensitive agriculture program? Evidence from a randomized controlled trial in Burkina Faso. | Low | Low | Low | Low | Come concern | Some concern | Low | Low | Low | Low | Some concern | Some concern |
| Jones et al. | 2019 | Women's empowerment and child nutrition: The role of intrinsic agency | Low | Low | Low | Low | Low | Low | Low | Low | Low | Low | Low | Low |
| Tewabe et al | 2020 | Women's Empowerment, Socioeconomic Status and Demographic Factors of Contraception in Amhara National Regional State, Ethiopia: A Sequential Analysis | Low | Low | Low | Low | Low | Low | Low | Low | Low | Low | Low | Low |
| Yaya et al. | 2020 | What does women’s empowerment have to do with malnutrition in Sub-Saharan Africa? Evidence from demographic and health surveys from 30 countries | Low | Low | Low | Low | Low | Low | Low | Low | Low | Low | Low | Low |
| Abreha et al. | 2020 | Associations between women's empowerment and children's health status in Ethiopia. | Low | Low | Low | Low | Low | Low | Low | Low | Low | Low | Low | Low |
| Anderson et al. | 2020 | Influence of Women's Empowerment on Place of Delivery in North Eastern and Western Kenya: A Cross-sectional Analysis of the Kenya Demographic Health Survey. | Low | Low | Low | Low | Low | Low | Low | Low | Low | Low | Low | Low |
| Kazembe | 2020 | Women Empowerment in Namibia: Measurement, Determinants, and Geographical Disparities. | Low | Low | Low | Low | Low | Low | Low | Low | Low | Low | Low | Low |
| Ewerling et al. | 2020 | The impact of Women’s empowerment on their children’s early development in 26 African countries | Low | Low | Low | Low | Low | Low | Low | Low | Low | Low | Low | Low |
| Soharwardi and Ahmed | 2020 | Dimensions and Determinants of Women Empowerment in Developing Countries | Low | Low | Low | Low | Low | Low | Low | Low | Low | Low | Low | Low |
| Bengesai and Derera | 2021 | The Association Between Women Empowerment and Emotional Violence in Zimbabwe: A Cluster Analysis Approach | Low | Low | Low | Low | Low | Some concern | Low | Low | Low | Low | Low | Low |
| Dickson et al. | 2021 | Women empowerment and skilled birth attendance in sub-Saharan Africa: A multi-country analysis. | Low | Low | Low | Low | Low | Low | Low | Low | Low | Low | Low | Low |
| Ameyaw et al. | 2021 | Is improvement in indicators of women's empowerment associated with uptake of WHO recommended IPTp-SP levels in sub-Saharan Africa? A multilevel approach. | Low | Low | Low | Low | Low | Low | Low | Low | Low | Low | Low | Low |
| Bliznashka et al. | 2021 | Associations between women's empowerment and child development, growth, and nurturing care practices in sub-Saharan Africa: A cross-sectional analysis of demographic and health survey data. | Low | Low | Low | Low | Low | Low | Low | Low | Low | Low | Low | Low |
| Kareem et al. | 2021 | Temporal relationship between Women's empowerment and utilization of antenatal care services: lessons from four National Surveys in sub-Saharan Africa. | Low | Low | Low | Low | Low | Low | Low | Low | Low | Low | Low | Low |
| Porth et al. | 2021 | Women's Empowerment and Child Vaccination in Kenya: The Modifying Role of Wealth. | Low | Low | Low | Low | Low | Low | Low | Low | Low | Low | Low | Low |
| Mekonnen et al. | 2021 | Women's empowerment and child growth faltering in Ethiopia: evidence from the Demographic and Health Survey. | Low | Low | Low | Low | Low | Low | Low | Low | Low | Low | Low | Low |
| Ameyaw et al. | 2021 | Women's empowerment and female genital mutilation intention for daughters in Sierra Leone: a multilevel analysis. | Low | Low | Some concern | Some concern | Low | Low | Low | Low | Low | High | Low | Some concern |
| Dickson | 2021 | Women Empowerment and Skilled Birth Attendants among Women in Rural Ghana. | Low | Low | Low | Low | Low | Low | Low | Low | Low | Low | Low | Low |
| Castro Lopes et al. | 2021 | Socio-economic, demographic, and behavioural determinants of women's empowerment in Mozambique. | Low | Low | Low | Low | Low | Low | Low | Low | Low | Low | Low | Low |
| Muluneh et al. | 2021 | The Effect of Women's Empowerment in the Utilisation of Family Planning in Western Ethiopia: A Structural Equation Modelling Approach. | Low | Low | Low | Low | Low | Low | Low | Low | Low | Low | Low | Low |
| Ameyaw et al. | 2021 | Do women empowerment indicators predict receipt of quality antenatal care in Cameroon? Evidence from a nationwide survey. | Low | Low | Low | Low | Low | Low | Low | Low | Low | Low | Low | Low |
| Adokiya et al. | 2021 | Women's autonomy and modern contraceptive use in Ghana: a secondary analysis of data from the 2014 Ghana Demographic and Health Survey. | Low | Low | Low | Low | Low | Low | Low | Low | Low | Low | Low | Low |
| Some et al. | 2021 | Empowerment and use of modern contraceptive methods among married women in Burkina Faso: a multilevel analysis. | Low | Low | Low | Low | Low | Low | Low | Low | Low | Some concern | Low | Low |
| Dadzie et al. | 2021 | Women empowerment and minimum daily meal frequency among infants and young children in Ghana: analysis of Ghana demographic and health survey. | Low | Low | Low | Low | Low | Low | Low | Low | Low | Low | Low | Low |
| Singh et al. | 2021 | The association of empowerment measures with maternal, child and family planning outcomes in Plateau State Nigeria by urban‒rural residence. | Low | Low | Low | Low | Low | Low | Low | Low | Low | Low | Low | Low |
| Mganga et al. | 2021 | Development of a Women’s Empowerment Index for Tanzania from the Demographic and Health Surveys of 2004–05, 2010, and 2015–16. | Low | Low | Low | Low | Low | Low | Low | Low | Low | Low | Low | Low |
| Zegeye et al. | 2022 | Women's decision-making power and knowledge of prevention of mother to child transmission of HIV in sub-Saharan Africa. | Low | Low | Low | Low | Low | Low | Low | Low | Low | Low | Low | Low |
| Lewis et al. | 2022 | Associations between women's empowerment, care seeking, and quality of malaria care for children: A cross-sectional analysis of demographic and health surveys in 16 sub-Saharan African countries. | Low | Low | Low | Low | Low | Low | Low | Low | Low | Low | Low | Low |
| Mokam and Zamo Akono | 2022 | The association between women's empowerment and reproductive health care utilization in Cameroon. | Low | Low | Low | Low | Low | Low | Low | Low | Low | Low | Low | Low |
| Leasure et al. | 2022 | Women's empowerment and attitudes towards female genital mutilation abandonment in Nigeria: A cross-sectional analysis of the Nigeria demographic health survey. | Low | Low | Low | Low | Some concern | Low | Low | Low | Low | High | Low | Come concern |
| Kawuki et al. | 2022 | Women empowerment and health insurance utilisation in Rwanda: a nationwide cross-sectional survey. | Some concern | Low | Low | Low | Low | Low | Low | Low | Low | High | Low | Some concern |
| Adde et al. | 2022 | Women's empowerment indicators and short- and long-acting contraceptive method use: evidence from DHS from 11 countries. | Low | Low | Low | Low | Low | Low | Low | Low | Low | Low | Low | Low |
| Komakech et al. | 2022 | The associations between women's empowerment measures, child growth and dietary diversity: Findings from an analysis of demographic and health surveys of seven countries in Eastern Africa. | Low | Low | Low | Low | Low | Low | Low | Low | Low | Low | Low | Low |
| Ntoimo et al. | 2022 | Influence of women's empowerment indices on the utilization of skilled maternity care: evidence from rural Nigeria. | Low | Low | Low | Low | Low | Low | Low | Low | Low | Low | Low | Low |
| Seidu et al. | 2022 | Women empowerment indicators and uptake of child health services in sub-Saharan Africa: a multilevel analysis using cross-sectional data from 26 countries. | Low | Low | Low | Low | Low | Low | Low | Low | Low | Low | Low | Low |
| Sserwanja et al. | 2022 | Dimensions of women empowerment on access to antenatal care in Uganda: A further analysis of the Uganda demographic health survey 2016. | Low | Low | Low | Low | Low | Low | Low | Low | Low | Low | Low | Low |
| Castro Lopes et al. | 2022 | How women’s Empowerment Influences Fertility-related Outcomes and Contraceptive Practices: A cross-sectional Study in Mozambique. | Low | Low | Low | Low | Low | Low | Low | Low | Low | Low | Low | Low |
| Arthur-Holmes et al. | 2023 | Safer Sex Negotiation Among Ghanaian Women in Sexual Unions: Does Women's Household Decision-Making Capacity Matter? | Low | Low | Low | Some concern | Low | Low | Low | Low | Low | Low | Low | Low |
| Zegeye et al. | 2023 | Association between women's household decision-making autonomy and health insurance enrolment in Sub-Saharan Africa. | Low | Low | Low | Low | Low | Low | Low | Low | Low | Low | Some concern | Low |
| Wilunda et al. | 2023 | Association of women's empowerment with anaemia and haemoglobin concentration in children in sub-Saharan Africa: A multilevel analysis. | Low | Low | Low | Low | Low | Low | Low | Low | Low | Low | Low | Low |
| Anik at al. | 2023 | Association of spousal violence and women's empowerment status among the rural women of sub-Saharan Africa. | Low | Low | Low | Low | Low | Low | Low | Low | Low | Low | Low | Low |
| Schierl et al. | 2023 | The Association of Women's Empowerment with HIV-Related Indicators: A Pooled Analysis of Demographic and Health Surveys in Sub-Saharan Africa. | Low | Low | Low | Low | Low | Low | Low | Low | Low | Low | Low | Low |
| Christian et al. | 2023 | Women's empowerment, children's nutritional status, and the mediating role of household headship structure: Evidence from sub-Saharan Africa. | Low | Low | Low | Low | Low | Low | Low | Low | Low | Low | Low | Low |
| Habte et al. | 2023 | Women empowerment domains and unmet need for contraception among married and cohabiting fecund women in Sub-Saharan Africa: A multilevel analysis based on gender role framework. | Low | Low | Low | Low | Low | Low | Low | Low | Low | Low | Low | Low |
| Tagang and Rwenge | 2023 | Women's autonomy and fertility in Chad. | Low | Low | Low | Low | Low | Low | Low | Low | Low | Low | Low | Low |
| Sey-Sawo et al. | 2023 | Women's empowerment and nutritional status of children in the Gambia: further analysis of the 2020 Gambia demographic and health survey. | Low | Low | Low | Low | Low | Low | Low | Low | Low | Low | Low | Low |
| Odwe et al. | 2023 | Women's empowerment and uptake of sulfadoxine-pyrimethamine for intermittent preventive treatment of malaria during pregnancy: results from a cross-sectional baseline survey in the Lake endemic region, Kenya. | Low | Low | Low | Low | Low | Low | Low | Low | Low | Low | Low | Low |
| Amoah et al. | 2023 | Influence of women empowerment on childhood (12-23 months) immunization coverage: Recent evidence from 17 sub-Saharan African countries. | Low | Low | Low | Low | Low | Low | Low | Low | Low | Low | Low | Low |
| Zhou et al. | 2023 | Influences of women empowerment indices on demand for childcare services: Evidence from the Nigeria Demographic and Health Surveys. | Low | Low | Low | Low | Low | Low | Low | Low | Low | Low | Low | Low |
| Sserwanja et al. | 2023 | Women empowerment indices and utilization of health facilities during childbirth: evidence from the 2019 Sierra Leone demographic health survey. | Low | Low | Low | Low | Low | Low | Low | Low | Low | Low | Low | Low |
| Cardona et al. | 2024 | Measuring Women's Economic Empowerment in the Democratic Republic of Congo, Kenya, Nigeria, and Burkina Faso | Low | Low | Low | Low | Low | Low | Low | Low | Low | Low | Low | Low |
| Aboagye et al. | 2024 | Does women's empowerment and socio-economic status predict adequacy of antenatal care in sub-Saharan Africa? | Low | Low | Low | Low | Low | Low | Low | Low | Low | Low | Low | Low |
| Donkoh et al. | 2024 | Association between the survey-based women's empowerment index (SWPER) and intimate partner violence in sub-Saharan Africa. | Low | Low | Low | Low | Low | Low | Low | Low | Low | Low | Low | Low |
| Aboagye et al. | 2024 | Can women empowerment boost dietary diversity among children aged 6-23 months in sub-Saharan Africa? | Low | Low | Low | Low | Low | Low | Low | Low | Low | Low | Low | Low |
| Salihu et al. | 2024 | Spatial Heterogeneity and association between the survey-based Women's Empowerment Index (SWPER) and unmet need for birth spacing in sub-Saharan Africa. | Low | Low | Low | Low | Low | Low | Low | Low | Low | Low | Low | Low |
| Dadzie et al. | 2024 | Association between women's empowerment and fertility preferences in Ghana. | Low | Low | Low | Low | Low | Low | Low | Low | Low | Low | Low | Low |
| Bamusi et al. | 2024 | Women's empowerment and its influence on the uptake of breast cancer screening in Tanzania: an analysis of 2022 Tanzania demographic health survey data. | Low | Low | Low | Low | Low | Low | Low | Low | Low | Low | Low | Low |
| Nibogore and Eryurt | 2024 | Women's Empowerment and Infant Mortality: Evidence from Rwanda. | Low | Low | Low | Low | Low | Low | Low | Low | Low | Low | Low | Low |
| Eom et al. | 2024 | Women's empowerment and child anthropometric failures across 28 sub-Saharan African countries: A cross-level interaction by Gender Inequality Index. | Low | Low | Low | Low | Low | Low | Low | Low | Low | Low | Low | Low |
| Makongote et al. | 2024 | Women's Empowerment and Associated Factors in Kinshasa, Democratic Republic of Congo: A Secondary Data Analysis of the Performance Monitoring Assessment Survey. | Low | Low | Low | Low | Low | Low | Low | Low | Low | Low | Low | Low |
| Achana et al. | 2024 | Women's autonomy, neonatal, infant and under-five mortality in the Upper East Region of Ghana. | Low | Low | Low | Low | Low | Low | Low | Low | Low | Low | Low | Low |
| Baye et al. | 2024 | Empowering women can improve child dietary diversity in Ethiopia. | Low | Low | Low | Low | Some concern | Low | Low | Low | Low | Low | Low | Low |
| Wassie et al. | 2024 | Women empowerment and childhood stunting: evidence from rural northwest Ethiopia. | Low | Low | Low | Low | Low | Low | Low | Low | Low | Low | Low | Low |
| Hellwig et al. | 2024 | Association between women’s empowerment and demand for family planning satisfied among Christians and Muslims in multireligious African countries | Low | Low | Low | Low | Low | Low | Low | Low | Low | Low | Low | Low |
| Salcedo et al. | 2025 | Measuring Multidimensional Women's Empowerment with Data from Partnered Women Across 45 Low- And Middle-Income Countries | Low | Low | Low | Low | Low | Low | Low | Low | Low | Low | Low | Low |
| Eom et al. | 2025 | Individual- and community-level women's empowerment and complete use of maternal healthcare services: A multilevel analysis of 34 sub-Saharan African countries | Low | Low | Low | Low | Low | Low | Low | Low | Low | Low | Low | Low |
| Yu et al. | 2025 | Women's Empowerment in Zimbabwe: Examining the Role of Educational Reform | Low | Low | Low | Low | Low | Some concern | Low | Low | Low | Low | Low | Low |
| Ayebeng et al. | 2025 | Cohabitation in sub-Saharan Africa: Does women empowerment matter? Insights from the demographic and health survey. | Low | Low | Low | Low | Low | Low | Low | Low | Low | Low | Low | Low |
| Aboagye et al. | 2025 | Association between the Survey-based Women's Empowerment (SWPER) index and barriers to healthcare in sub-Saharan Africa. | Low | Low | Low | Low | Low | Low | Low | Low | Low | Low | Low | Low |
| Abebe et al. | 2025 | The association of women's empowerment dimensions and antenatal care utilization in Ethiopia; facility based cross-sectional study. | Low | Low | Low | Low | Low | Low | Low | Low | Low | Low | Low | Low |
| Shibre et al. | 2025 | Level of and trends in women's empowerment inequalities in antenatal care services in Ethiopia: further analysis of the Ethiopia demographic and health surveys, 2000-16. | Low | Low | Low | Low | Low | Low | Low | Low | Low | Low | Low | Low |
| Ahmed et al. | 2025 | Do empowerments influence experiencing intimate partner violence (IPV)? A multi-continental study of women across low and lower-middle-income countries. | Low | Low | Low | Low | Low | Low | Low | Low | Low | Low | Low | Low |

Notes

D1: Did the study address a clearly focused issue?

D2: Did the authors use an appropriate method to answer their question?

D3: Were the subjects recruited in an acceptable way?

D4: Were the measures accurately measured to reduce bias?

D5: Were the data collected in a way that addressed the research issue?

D6: Did the study have enough participants to minimise the play of chance?

D7: How are the results presented and what is the main result?

D8: Was the data analysis sufficiently rigorous?

D9: Is there a clear statement of findings?

D10: Can the results be applied to the local population?

D11: How valuable is the research?

Table S3: Characteristics of the 98 publications included in this review

| **Author** | **Year** | **Title** | **Study location** | **Data** | **Measure of women empowerment** |
| --- | --- | --- | --- | --- | --- |
| Hindin and Muntifering | 2011 | Women's autonomy and timing of most recent sexual intercourse in sub-Saharan Africa: A multi-country analysis | 6 African countries | DHS | **1 Domain**  ***Decision making autonomy:*** Person who decides on respondents’ health care, on larger household purchases, on visit to family or relatives & on household purchases for daily needs. |
| Upadhyay and Karasek | 2012 | Women’s Empowerment and Ideal Family Size: An Examination of Empowerment Measures in Sun-Saharan Africa | 4 African Countries | DHS | **3 Indicators**  ***Household decision making:*** Health care, major household purchases, daily household purchases, visit family.  ***Attitudes toward wife beating:*** Goes out without permission, neglect children, argues with him, burns food &  ***Attitudes toward refusing sex:*** When husband has STI, has other women, has recently given birth, tired or not in mood. |
| Do and Kurimoto | 2012 | Women's empowerment and choice of contraceptive methods in selected African countries. | Namibia, Zambia, Ghana and Uganda | 2006-2008 DHS | **4 Dimensions**  ***Economic:*** Income contribution relative to husband, Decision on how each partners income would be used and decision about major and daily household purchases.  ***Sociocultural:*** Who decided whether they could visit their family and relatives.  ***Familial and Interpersonal:*** Domestic decision making, Control over sexual relations, Marriage, Fertility, Contraception, Health-seeking behaviour and  **Attitudes regarding domestic violence**. |
| Kwagala et al. | 2013 | Empowerment, partner's behaviours and intimate partner physical violence among married women in Uganda. | Uganda | 2011 DHS | **3 Domains**  ***Economic:*** Ownership of a property  ***Decision making:*** Person who decides on respondent’s own income, husband income spending, respondent’s health care, house earning and household purchases and visiting family and  ***Attitudes justifying violence:*** Neglect of a child, burning of food, arguing with husband, refuse sex, going out without permission |
| Jennings et al. | 2014 | Women's empowerment and male involvement in antenatal care: analyses of Demographic and Health Surveys (DHS) in selected African countries. | Selected African countries | 2010-2011 DHS | **3 Dimensions**  ***Economic:*** Income generation, control over finance, ***Socio-familial:*** Freedom of mobility, control of own health, free of domestic violence and  ***Legal:*** Rights over land and houses |
| Corroon et al. | 2014 | The role of gender empowerment on reproductive health outcomes in urban Nigeria. | Nigeria - URBAN | Measurement, Learning and Evaluation Project (MLE) part of Nigerian Urban Reproductive Health Initiative (NU- RHI) | **4 Dimensions:**  ***Attitudes towards domestic violence:*** If goes out, neglects children, argues with him, refuses sex, burn food, suspected of unfaithful, refuses to have another child.  ***Partner prohibition:*** Work outside home, visit from people, visit friends, visit family, using phone, using contraceptives.  ***Decision-making:*** Large HH purchases, small purchases, deciding when to visit family, seek medical care.  ***Economic Freedom:*** Access to money of her own. |
| Solanke | 2015 | Marriage Age, Fertility Behaviour, and Women's Empowerment in Nigeria | Nigeria | 2013 DHS | **2 domains**:  ***Women autonomy:*** Person who decides on respondent’s health care, on larger HH purchases, on visit to family or relative and  ***Education:*** Education level |
| Na et al. | 2015 | Association between women's empowerment and infant and child feeding practices in sub-Saharan Africa: an analysis of Demographic and Health Surveys. | SSA Countries | 2010-2013 DHS | **3 Dimensions:**  ***Economic:*** Income generation, control over finance, ***Socio-familial:*** Freedom of mobility, control of own health, free of domestic violence and  ***Legal:*** Rights over land and houses |
| Shimamoto and Gipson | 2015 | The relationship of women's status and empowerment with skilled birth attendant use in Senegal and Tanzania. | Tanzania and Senegal | 2010 DHS | **4 Domains:**  ***Household decision-making powe****r:* Own health care, major household purchases, and visits to family or relatives,  ***Perceptions of gender norms against violence:*** If she goes out without telling him, neglects the children, argues with him, refuses to have sex with him, or burns the food,  ***Perceptions of gender norms for sex negotiation:*** If the respondent can refuse having sex or can ask her partner to use a condom and  ***Age at first marriage*** |
| Ibrahim et al. | 2015 | The Influences of Women's Empowerment on Reproductive Health outcomes: A comparative study of Nigeria and Uttar Pradesh State (India). | India and Nigeria | *Nigerian DHS 2008 & Indian National Family Health Survey (NFHS-3)* | **3 Dimensions:**  ***Decision making:*** Respondents use of contraceptives, health care, large household purchases, daily household purchases, visit to family,  ***Attitude toward domestic violence:***  Wife beating justified- goes out, neglect children, refuse sex, argues with him, burn food and  ***Attitudes towards refusing sexual intercourse:*** If husband has STI, has intercourse with other women, tired not in mood. |
| Asaolu et al. | 2016 | Association between Measures of Women’s Empowerment and Use of Modern Contraceptives: An Analysis of Nigeria’s Demographic and Health Surveys. | Nigeria | DHS | **1 Domain:**  ***Decision making variable:*** Decision maker on visiting family, health care, & household purchases. |
| Blackstone | 2017 | Women’s Empowerment, Household Status and Contraception Use in Ghana. | Ghana | 2014 DHS | **3 Indicators**:  ***Participation in decision making:*** Health care, major household purchases, spending respondent money & visiting relatives,  ***Attitudes toward wife beating:*** Burn food, if she withholds information from him, neglect children, she argues with him, if refuses to have sex with him, &  ***Women’s household status:*** Work status, relationship HH head, control over earning and land ownership. |
| Lan and Tavrow | 2017 | Composite measures of women's empowerment and their association with maternal mortality in low-income countries. | 44 Low Income Countries | 2016 World Bank Annual Report | **3 Indices**:  Gender Gap Index,  Gender Equity Index (GEI) and  Social Institutions and Gender Index (SIGI) |
| Tiruneh et al. | 2017 | Women's autonomy and maternal healthcare service utilization in Ethiopia. | Ethiopia | 2005-2011 DHS | **2 Indicators:**  ***Decision makin****g:* Woman’s health, major purchases, visits to friends or family and  ***Attitude towards wife beating:*** She goes out without telling him, she neglects the children, she argues with him, she refuses to have sexual intercourse with him, and she does not cook food properly. |
| Alaofe et al. | 2017 | Association Between Women's Empowerment and Maternal and Child Nutrition in Kalale District of Northern Benin. | Benin | 2014 Solar Market Garden Study | **6 Dimensions**:  ***Leadership:*** Sense of self and confidence,  ***Decision making:*** Decision maker on members of household's health care, regarding decisions on kids’ schooling & regarding whether to have another child or not,  ***Mobility:*** Permission to market, to health centre, to visit friends,  ***Economic securit****y:* Afford fruits and veg, own clothes own beauty purchase,  ***Male involvemen****t:*  Help from adult male and  ***Nonfamily groups:*** Group member. |
| Bayissa et al. | 2017 | The Multidimensional Nature of Women’s Empowerment: Beyond the Economic Approach | Ethiopia | Primary data | **6 dimensions:**  ***Economic:*** Income generating by women, asset ownership,  ***Familial:*** Decision making power,  ***Phycologica****l:* Self esteem indicators,  ***Legal:*** Knowledge of women legal rights,  ***Political:***  Political representation, participation in public protests/campaigning &  ***Sociocultural:*** Equal effectiveness of boys and girls at school, reports on female genital mutilation, changing socio-cultural things in the community. |
| Asaolu et al. | 2018 | Measuring Women’s Empowerment in Sub-Saharan Africa: Exploratory and Confirmatory Factor Analyses of the Demographic and Health Surveys. | 19 countries Central Africa: Southern Africa: West Africa | DHS | **4 factors**  ***Education:*** Literacy, highest education level & spousal difference in educational attainment,  ***Socio-cultural:*** Attitude towards violence, HH decision making, life course indicator, land & home ownership, ***Economic:*** *O*ccupation, type of earning, seasonality of occupation & income ratio &  ***Access to health care:*** Women’s ability to refuse sex or ask a partner to use condoms. |
| Yaya et al. | 2018 | Women’s Empowerment as an Enabling Factor of Contraceptive Use in Sub-Saharan Africa: A Multilevel Analysis of Cross-sectional Surveys of 32 Countries. | Central, Eastern, Southern: Western countries: | DHS | **4 Indicators**  **Labour force participation:** Employment status,  A**cceptance of wife beating:** Beating justified if wife neglects children, refuse to have sex, burn food & goes out without telling the husband,  **Knowledge level:** Education level, listen to the radio, reads a newspaper and watches television and  **Decision making:** Decision on respondent’s healthcare, house earning, household purchases and visiting family members. |
| Ford et al. | 2018 | Women's decision-making and uptake of services to prevent mother-to-child HIV transmission in Zambia. | Zambia | PMTCT Household Survey | **1 Variable**  **Decision making autonomy:** Healthcare, large household purchases, schooling children and healthcare of children. |
| Voronca et al. | 2018 | Relationship between empowerment and wealth: trends and predictors in Kenya between 2003 and 2008-2009. | Kenya | 2003 and 2008–2009 DHS | **2 Measures**  **Participation in decision making:** Major household purchases, purchase of daily household needs, visiting female partner’s family or relatives, money female partner earns, and own heath care/how many children to have and  **Attitudes towards domestic violence:** When female partner burns the food, argues with male partner, goes out without telling the male partner, neglects the children, refuses sexual intercourse with the male partner. |
| Miedema et al. | 2018 | Women’s empowerment in East Africa: Development of a Cross-country Comparable Measure. | East Africa: (Ethiopia, Kenya, Rwanda, Tanzania, and Uganda) | DHS | **3 Domains**  **Human and social assets:** Schooling attainment, age at first sex, cohabitation, birth, spousal age difference, spousal schooling difference, spousal earning difference, first sex at marriage, work for cash or in-kind,  **Gender beliefs/ attitudes:** Beating justified if- goes out, neglect children, argues, refuses sex, burn food, had sex outside marriage, wife justified refusing sex if husband have sex with other women, to ask for condom if husband has STI, and  **Household & decision making:** Decision to visit family/relatives, health care, major house purchases and husband/partners earning, respondents earning. |
| Ebrahim and Atteraya | 2019 | Women's Household Decision-Making and Intimate Partner Violence in Ethiopia | Ethiopia | 2016 DHS | **1 Domain**  **Decision making Autonomy:** Person who decides on respondent’s health care, on larger household purchases, on visit to family or relatives & on household finances. |
| Atake and Gnakou-Ali | 2019 | Women’s Empowerment and Fertility Preferences in High Fertility Countries in Sub-Saharan Africa. | Burkina Faso, Mali, Niger, and Chad | DHS | **3 Dimensions**  **Family:** Age at 1st birth, age at first sex, decision on household purchases, decision on contraceptive use, visiting family, respondent’s health care, heard of FP on radio, on TV, on Newspaper,  **Economic:** Working, type of earning, own a house, own land) and  **Sociocultural:** Education, frequency of listening to radio, reading a newspaper. |
| Mengo et al. | 2019 | Marital Rape and HIV Risk in Uganda: The Impact of Women's Empowerment Factors. | Uganda | 2011 DHS | **2 Indicators**  Labour force participation Respondents currently working,  **Decision making** large household purchase, health care, visit family and how to spend respondent earning. |
| Chol et al. | 2019 | Women's autonomy and utilisation of maternal healthcare services in 31 Sub-Saharan African countries: results from the demographic and health surveys, 2010-2016. | 31 SSA countries | DHS | **2 Areas**  **Decision-making:** Decision making on spending of household income and  **Attitude towards physical abuse:** Justified in beating his wife if she goes out without telling him; neglects the children; argues with him; or burns the food. |
| McKenna et al. | 2019 | Women's decision-making power and undernutrition in their children under age five in the Democratic Republic of the Congo: A cross-sectional study. | DRC | 2013-14 DHS | **1 Indicator**  **Decision making power:** Person who decides on respondent’s own income, husband income spending, respondent’s health care, house earning and household purchases and visiting family. |
| Heckert et al. | 2019 | Is women's empowerment a pathway to improving child nutrition outcomes in a nutrition-sensitive agriculture program? Evidence from a randomized controlled trial in Burkina Faso. | Burkina Faso | HH Survey | **4 Domains**  **Purchasing decisions:** To purchase small quantities of food, larger quantities of food, clothing for herself, medication for herself, and special foods for her children,  **Healthcare decisions:** She contributed to decisions to consult a doctor, when she was pregnant and what to do when a child was sick,  **Family planning decisions:** She contributed to decisions to use a contraceptive method and have another child, and  **Spousal communication:** Domestic activities, expenses, child health, nutrition, personal health. |
| Jones et al. | 2019 | Women's empowerment and child nutrition: The role of intrinsic agency | 5 SSA Countries | 2011-2016 DHS | **3 Domains**  **Human and social assets:** Age at first sex, cohabitation, birth,  G**ender beliefs/ attitude**s: Beating justified if- goes out, neglect children, argues, refuses sex, burn food and  **Household & decision making:** Decision to visit family/relatives, health care, major HH purchases and respondents earning. |
| Tewabe et al | 2020 | Women's Empowerment, Socioeconomic Status and Demographic Factors of Contraception in Amhara National Regional State, Ethiopia: A Sequential Analysis | Ethiopia | Primary data | **4 Domains**  **Mobility freedom:** Decision to visit family/relatives, health care & social affairs,  **Financial Autonomy:** Earn income, own saving, spending cash earnings & husband income,  **Access to resources:** Ownership of major durable items, land & house and  **Free from spousal violence:** Ever physical, emotional, economic & sexual violence. |
| Yaya et al. | 2020 | What does women’s empowerment have to do with malnutrition in Sub-Saharan Africa? Evidence from demographic and health surveys from 30 countries | 30 countries | 2011 to 2017 DHS | **2 Indicators**  **Decision making:** Health care, major purchases & visiting family and  **Justification for violence:** Burning food, arguing with him, going out without telling him, neglecting the children and refusing to have sexual intercourse with him, |
| Abreha et al. | 2020 | Associations between women's empowerment and children's health status in Ethiopia. | Ethiopia | 2016 DHS | **5 Dimensions**  **Decision making:** Decision on respondent’s healthcare, large household purchases, money husband earn, and visiting family or relatives,  **Attitudes towards wife beating:** Beating justified if wife goes out without telling him, she neglects children, she argues with him, refuses sex & burn food,  **Barriers to health car**e: Getting permission, getting money for treatment, distance to facility, not wanting to go alone,  **Socioeconomic status:** Highest education level, frequency of reading newspaper, frequency of watching TV, frequency of internet use, has a bank account, own a mobile phone, employment and  **Assess ownership:** Own a house and own land. |
| Anderson et al. | 2020 | Influence of Women's Empowerment on Place of Delivery in North Eastern and Western Kenya: A Cross-sectional Analysis of the Kenya Demographic Health Survey. | Kenya | 2014 DHS | **4 factors**  **Education:** Literacy, highest education level & spousal difference in educational attainment,  **Socio-cultural:** Attitude towards violence, household decision making, life course indicator, land & home ownership,  **Economic:** Occupation, type of earning, seasonality of occupation & income ratio &  A**ccess to health care:** Women’s ability to refuse sex or ask a partner to use condoms. |
| Kazembe | 2020 | Women Empowerment in Namibia: Measurement, Determinants, and Geographical Disparities. | Namibia | DHS | **3 Dimensions**  **Economic:** Respondent employed, deciding on spending money, amount of earning,  **Socio-cultural/Psychological:** Wife beating justified- goes out, neglect children, refuse sex, argues with him, burn food) and  **Familial/Interpersonal:** Decision making on health care, major household purchases, daily household needs, visiting relatives, money husband earns, refuse to have sex-husband has STI, have other women, if tired/not in the mood, no condom if husband has STD, decision maker on contraceptive use, husband knows. |
| Ewerling et al. | 2020 | The impact of Women’s empowerment on their children’s early development in 26 African countries | 26 African Countries | Multiple Indicator Cluster Survey (MICS) & DHS | **3 SWPER domains**  **Attitude to violence:** Beating justified wife neglects children, refuse to have sex, burn food,  **Decision making:** Decision on health care, & purchase of HH goods and  **Social independence:** Worked in last 12 months, age at cohabitation, age at first birth, & age difference between wife and husband. |
| Soharwardi and Ahmed | 2020 | Dimensions and Determinants of Women Empowerment in Developing Countries | Developing countries | DHS | **5 Dimensions**  **Work status:** Currently working, employment status,  **Awareness:** Watching TV, reading newspaper or magazine, listening to the radio, heard about family planning on the radio, TV, from newspapers,  **Decision Making:** To spend husband earning, on health care, large household purchases, visits to family or relatives,  **Self Esteem:** Goes out without telling husband, neglect the children, argue with husband, refuse to have sex, burn the food,  **Self Confidence:** Getting medical help for self, want to go alone, getting money for treatment. |
| Bengesai and Derera | 2021 | The Association Between Women Empowerment and Emotional Violence in Zimbabwe: A Cluster Analysis Approach | Zimbabwe | 2015 DHS | **6 Domains**  **Education and literacy:** Highest education, ability to read a sentence,  **Economic development:** Currently working, types of earnings, Ownership of land, ownership of a house,  **Attitudes toward wife beating:** Wife burns food, neglect children, refuses sex and argues with husband,  **Economic decision-making:** Decision on respondent’s earning, on husbands earning, on household purchases,  **Mobility decision making:** Decision on visiting family, visiting health care.  **Health decision making:** Decision on contraceptive use, on personal of family health and on using a condom if husband has STI. |
| Dickson et al. | 2021 | Women empowerment and skilled birth attendance in sub-Saharan Africa: A multi-country analysis. | 29 countries in SSA | DHS | **4 Elements**  **Labour force:** Working, not working,  **Acceptance of Wife beating:** Neglect of a child, burning of food, arguing with husband, refuse sex, going out without permission,  **Decision making power:** Person who decides on respondent’s health care, house earning and household purchases, on visit to family or relatives,  **Knowledge level:** Listening to radio, reading newspaper/magazine, watching TV and educational level. |
| Ameyaw et al. | 2021 | Is improvement in indicators of women's empowerment associated with uptake of WHO recommended IPTp-SP levels in sub-Saharan Africa? A multilevel approach. | 20 SSA Countries | DHS | **4 Indicators**  **Knowledge:** Education attainment, exposure to radio, TV & newspaper,  **Reason for domestic violence:** Neglect children, burning food, declining sex, arguing with partner and visiting without permission,  **Decision making:** Decision to visit family/relatives, health care, household purchases and household earning and  **Employment status:** Engaging in paid job. |
| Bliznashka et al. | 2021 | Associations between women's empowerment and child development, growth, and nurturing care practices in sub-Saharan Africa: A cross-sectional analysis of demographic and health survey data. | 9 Sub-Saharan Africa | DHS | **3 dimensions**  **Access to and control over resources:** Seasonality, income relative to partners,  **Decision making:** Decision to visit family/relatives, health care, major household purchases and husband/partners earning, and  **Attitudes towards wife beating:** If goes without telling husband, neglect children, refuse sex. |
| Kareem et al. | 2021 | Temporal relationship between Women's empowerment and utilization of antenatal care services: lessons from four National Surveys in sub-Saharan Africa. | 4 Sub-Saharan Africa | DHS | **8 Components**  **Labour force participation:** Women worked in the last 12 months, who does woman work for? Type of earnings from woman's work, Type of occupation, Work all years,  **Disagrees with wife beating:** Goes out without telling husband, neglects the children, argue with husband, refuses to have sex with husband, burns food),  **Health decision making power:** Permission to get medical help, getting money for treatment, not wanting to go alone to health centres,  **Household decision making powe**r: Who decides on respondent health care? Who decides on large household purchases? Who decides on visit to family or relatives? Who usually decides on what to do with husband earnings?  **Gender norms for sex negotiation:** Can ask partner to use condom, can refuse sex,  **Knowledge level of survival:** Literacy, highest level of education,  **Exposure to mass media:** Newspaper, tv and radio), use of internet, have a bank account, own a phone or/and for transaction,  **Ownership of Assets:** Own a house, own land,  **Family planning knowledge:** Unmet need, heard family planning on radio, tv, print media or text message, Knowledge about contraceptive methods. |
| Porth et al. | 2021 | Women's Empowerment and Child Vaccination in Kenya: The Modifying Role of Wealth. | Kenya | 2014 DHS | **3 Domains**  **Human and social assets:** Schooling attainment, age at first sex, cohabitation, birth, spousal age difference, spousal schooling difference, spousal earning difference, first sex at marriage, work for cash or in-kind,  G**ender beliefs/attitudes:** Beating justified if- goes out, neglect children, argues, refuses sex, burn food, had sex outside marriage, wife justified refusing sex if husband have sex with other women, to ask for condom if husband has STI, and  H**ousehold & decision making:** Decision to visit family/relatives, health care, major household purchases and husband/partners earning, respondents earning. |
| Mekonnen et al. | 2021 | Women's empowerment and child growth faltering in Ethiopia: evidence from the Demographic and Health Survey. | Ethiopia | 2016 DHS | **3 SWPER domains**  **Attitude to violence:** Beating justified wife neglects children, refuse to have sex, burn food,  **Decision making:** Decision on health care, & purchase of HH goods and  **Social independence:** Worked in last 12 months, age at cohabitation, age at first birth, & age difference between wife and husband. |
| Ameyaw et al. | 2021 | Women's empowerment and female genital mutilation intention for daughters in Sierra Leone: a multilevel analysis. | Sierra Leone | 2013 DHS | **4 Indicators**  **Labour force participation:** Employment status,  A**cceptance of wife beating:** Beating justified if wife neglects children, refuse to have sex, burn food & goes out without telling the husband,  **Knowledge level:** Education level, listen to the radio, reads a newspaper and watches television and  **Decision making:** Decision on respondent’s healthcare, house earning, household purchases and visiting family members. |
| Dickson | 2021 | Women Empowerment and Skilled Birth Attendants among Women in Rural Ghana. | Ghana - Rural | 2014 DHS | **4 Elements**  **Labour force:** Working, not working,  **Acceptance of wife beating:** Neglect of a child, burning of food, arguing with husband, refuse sex, going out without permission,  **Decision making power:** Person who decides on respondent’s health care, house earning and household purchases and visiting family and  **Knowledge level:** Listening to the radio, read newspaper, watch TV and educational level. |
| Castro Lopes et al. | 2021 | Socio-economic, demographic, and behavioural determinants of women's empowerment in Mozambique. | Mozambique | 2015 DHS | **3 Domains**  **Beliefs about violence:** Neglect of a child, burning of food, arguing with husband, refuse sex, going out without permission,  **Decision making:** Woman’s health care, large purchases, visit family and friend) and  **Control over sexuality and safe sex**: Ask partner for condom, refuse if partner has STI. |
| Muluneh et al. | 2021 | The Effect of Women's Empowerment in the Utilisation of Family Planning in Western Ethiopia: A Structural Equation Modelling Approach. | Ethiopia | Primary data | **2 Domains**  **Decision making power:**  On big household purchases, own health, going to public meetings and  **Attitude towards physical violence:** Neglect of a child, burning of food, arguing with husband, refuse sex, going out without permission. |
| Ameyaw et al. | 2021 | Do women empowerment indicators predict receipt of quality antenatal care in Cameroon? Evidence from a nationwide survey. | Cameroon | 2018 DHS | **4 Indicators**  **Labour force:** Working, not working,  A**cceptance of wife beating:** Neglect of a child, burning of food, arguing with husband, refuse sex, going out without permission,  D**ecision making powe**r: Person who decides on respondent’s health care, house earning and household purchases and visiting family and  **Knowledge level** Listening to the radio, read newspaper, watching TV and educational level. |
| Adokiya et al. | 2021 | Women's autonomy and modern contraceptive use in Ghana: a secondary analysis of data from the 2014 Ghana Demographic and Health Survey. | Ghana | 2014 DHS | **3 Variables**  Household decision-making: Person who decides on respondent’s health care, house earning and household purchases and visiting family,  Attitudes towards wife-beating: Neglect of a child, burning of food, arguing with husband, refuse sex, going out without permission and  Property ownership: Own a house, own land |
| Some et al. | 2021 | Empowerment and use of modern contraceptive methods among married women in Burkina Faso: a multilevel analysis. | Burkina Faso | 2010 DHS | **3 Components**  **Influence in family decisions:** Decide on their own health care, make major household purchases, and visit family or relatives,  **Attitudes toward domestic violence:** If she burns food, if she argues with him, if she goes out without telling him, if she neglects the children, and if she refuses to have sexual intercourse with him and  **Freedom of movement in public spaces:** Permission to go for treatment, money for treatment, distance to a health facility, and a desire to not go alone. |
| Dadzie et al. | 2021 | Women empowerment and minimum daily meal frequency among infants and young children in Ghana: analysis of Ghana demographic and health survey. | Ghana | 2014 DHS | **3 Dimensions**  **Economic empowerment:** Control over women’s income, decision making on large household purchases, women’s ability to work outside home,  **Socio-familial empowerment:** Decision making regarding family visits, women’s own health, and attitude towards domestic violence under five scenarios and  **Legal empowerment:** Women’s judicial and legislative entitlements over land and over house ownership. |
| Singh et al. | 2021 | The association of empowerment measures with maternal, child and family planning outcomes in Plateau State Nigeria by urban‒rural residence. | Nigeria | 2017 Nigeria Urban Reproductive Health Initiative | **4 variables**  **Household decision making:** On large household purchases, small household purchases, visit family, deciding when and where to seek medical health,  **Financial decision makin**g: Decide on money partner earns,  **Views on wife beating:** If she goes out, neglect children, argues with husband, refuses sex, burn food, suspects of being unfaithful & refuses to have another child and  **Prohibitions:** Working outside home, having visits from people, visiting friends, visiting family & using a mobile phone. |
| Mganga et al. | 2021 | Development of a Women’s Empowerment Index for Tanzania from the Demographic and Health Surveys of 2004–05, 2010, and 2015–16. | Tanzania | DHS | **Six domains**  **Attitude towards violence:** If she goes out without telling husband, neglect the children, argue with husband, refuse to have sex, burn the food,  **Decision making:** Respondents health care, large household purchases, what to do with husband' money, visit to family, how to spend respondents earning  **Social independence** Respondent own a phone, watch TV, read newspaper, type of earning & educational attainment,  **Age at critical events:** Age of respondent at first birth, age at first cohabitating,  **Access to health care:** Getting help permission for medical help, money for treatment, distance to a health facility, not wanting to go alone for medical help and  **Property ownership:** Own a house, land. |
| Zegeye et al. | 2022 | Women's decision-making power and knowledge of prevention of mother to child transmission of HIV in sub-Saharan Africa. | 24 SSA Countries | DHS | **1 Domain**  **Decision making power:** Decision to visit family/relatives, health care, & large household purchases. |
| Lewis et al. | 2022 | Associations between women's empowerment, care seeking, and quality of malaria care for children: A cross-sectional analysis of demographic and health surveys in 16 sub-Saharan African countries. | 16 SSA Countries | 2010 - 2018 DHS | **4 Dimensions**  **Education:** Literacy, educational level, spousal difference in education,  **Economic:** Work/labour force participation, legal status,  **Sociocultural:** Household decision making, attitude toward violence, age of cohabitation and first birth) and  **Health:** Negotiating sex, access to health care. |
| Mokam and Zamo Akono | 2022 | The association between women's empowerment and reproductive health care utilization in Cameroon. | Cameroon | 2018 DHS | **2 Indices**  **Economic:** Respondent worked, own land and own house and  **Decision making:** Expenditure of woman earning, partners income, large household purchases, health care and visit relatives. |
| Leasure et al. | 2022 | Women's empowerment and attitudes towards female genital mutilation abandonment in Nigeria: A cross-sectional analysis of the Nigeria demographic health survey. | Nigeria | 2018 DHS | **4 factors**  **Education:** Literacy, highest education level & spousal difference in educational attainment,  **Socio-cultural:** Attitude towards violence, household decision making, life course indicator, land & home ownership,  **Economic:** Occupation, type of earning, seasonality of occupation & income ratio &  A**ccess to health care:** Women’s ability to refuse sex or ask a partner to use condoms. |
| Kawuki et al. | 2022 | Women empowerment and health insurance utilisation in Rwanda: a nationwide cross-sectional survey. | Rwanda | 2020 DHS | **4 Indices**  **Exposure to media:** Women’s ability to have the opportunity to read a newspaper or a magazine, listen to the radio and watch TV,  **Decision making:** Women’s participation in making decisions regarding; their own health; large household purchases; visits to their family and control over family earnings,  **Economic empowerment:** Women’s owning of a house, land and the type of earning from her work and  **Sexual empowerment:** Women’s ability to refuse sex and ask a partner to use condoms. |
| Adde et al. | 2022 | Women's empowerment indicators and short- and long-acting contraceptive method use: evidence from DHS from 11 countries. | 11 SSA Countries | DHS | **4 Indicators**  **Labour force:** Respondent working, not working,  **Acceptance of wife beating:** Neglect of a child, burning of food, arguing with husband, refuse sex, going out without permission,  D**ecision making power:** Person who decides on respondent’s health care, house earning and household purchases and visiting family and  **Knowledge level:** Listening to the radio, read newspaper, watch TV and educational level. |
| Komakech et al. | 2022 | The associations between women's empowerment measures, child growth and dietary diversity: Findings from an analysis of demographic and health surveys of seven countries in Eastern Africa. | East Africa | DHS | **5 Measures**  **Economic:** Participation in income activity,  **Decision making:** Decision on respondent’s healthcare, large household purchases, money husband earn, and visiting family or relatives,  **Self esteem:** Women's perception and experience of violence and abuse, and  **Legal:** Ownership of property. |
| Ntoimo et al. | 2022 | Influence of women's empowerment indices on the utilization of skilled maternity care: evidence from rural Nigeria. | Nigeria / RURAL | Baseline HH Survey | **3 Domains**  **Influencer:** Attitudes towards wife beating,  **Resource:** Occupation, having a mobile phone etc. and  **Decision making** Health care, major purchases & visiting family. |
| Seidu et al. | 2022 | Women empowerment indicators and uptake of child health services in sub-Saharan Africa: a multilevel analysis using cross-sectional data from 26 countries. | 26 SSA Countries | DHS | **3 Components**  **Disagreement with reasons to justify wife beating:** Reasons such as burning food, neglect of children, refusal to have sex with husband, visitations without permission from husband and argument with husband,  **Decision-making power:** Visiting family members, respondent’s health care, house earning and household purchases and  **Knowledge level:** Education level, reading the newspaper, listening to the radio and watching television. |
| Sserwanja et al. | 2022 | Dimensions of women empowerment on access to antenatal care in Uganda: A further analysis of the Uganda demographic health survey 2016. | Uganda | 2016 DHS | **4 Indices**  **Decision making:** Their own health; large household purchases; visits to their family and control over their earnings,  **Economic empowerment:** Women's owning of a house, land and the type of earning from her work,  **Sexual empowerment:** Ability to refuse sex and ask a partner to use condoms and  **Exposure to media indices:** Opportunity to read a newspaper or a magazine, listen to the radio and watch television (TV). |
| Castro Lopes et al. | 2022 | How women’s Empowerment Influences Fertility-related Outcomes and Contraceptive Practices: A cross-sectional Study in Mozambique. | Mozambique | 2015 DHS | **3 domains**  **Decision making:** Visit family, large household purchases & women's health,  **Women justified beating:** If wife goes out, if neglects children, if argues with husband, refuses sex, burn food), and  **Control over sexuality and safe sex:** Ask husband condom, use condom if he is STI & can refuse. |
| Arthur-Holmes et al. | 2023 | Safer Sex Negotiation Among Ghanaian Women in Sexual Unions: Does Women's Household Decision-Making Capacity Matter? | Ghana | 2014 DHS | **1 domain**  **Decision making capacity:** Health care, household purchases, visit to family or relatives. |
| Zegeye et al. | 2023 | Association between women's household decision-making autonomy and health insurance enrollment in sub-saharan Africa. | 29 SSA countries | 2010 - 2020 DHS | **1 Domain**  Decision making: Person who decides on respondent’s health care, on larger household purchases & on visit to family or relatives. |
| Wilunda et al. | 2023 | Association of women's empowerment with anaemia and haemoglobin concentration in children in sub-Saharan Africa: A multilevel analysis. | 31 SSA countries | 2006 - 2019 DHS | **3 SWPER domains**  **Attitude to violence:** Beating justified wife neglects children, refuse to have sex, burn food,  **Decision making:** Decision on health care, & purchase of household goods and  **Social independence:** Worked in last 12 months, age at cohabitation, age at first birth, & age difference between wife and husband. |
| Anik at al. | 2023 | Association of spousal violence and women's empowerment status among the rural women of sub-Saharan Africa. | SSA Countries | 2015 - 2019 DHS | **4 Dimensions**  **Economic:** Labour force participation,  **Socio cultural status:** Women attitude towards violence,  **Health** Access to health care and  **Education** |
| Schierl et al. | 2023 | The Association of Women's Empowerment with HIV-Related Indicators: A Pooled Analysis of Demographic and Health Surveys in Sub-Saharan Africa. | 31 SSA countries | DHS | **3 SWPER domains**  **Attitude to violence:** Beating justified wife neglects children, refuse to have sex, burn food,  **Decision making:** Decision on health care, & purchase of HH goods and  **Social independence:** Worked in last 12 months, age at cohabitation, age at first birth, & age difference between wife and husband. |
| Christian et al. | 2023 | Women's empowerment, children's nutritional status, and the mediating role of household headship structure: Evidence from sub-Saharan Africa. | SSA Countries | DHS | **3 Domains**  **Decision making:** Woman own earning, husband earnings, visit to family, large HH purchases, daily needs purchases & respondents health care,  **Attitude to violence:** Beating justifies when she - burns food, argues with husband, goes out without permission, neglect children & refuses husband sex and  **Asset ownershi**p: Own house & own land. |
| Habte et al. | 2023 | Women empowerment domains and unmet need for contraception among married and cohabiting fecund women in Sub-Saharan Africa: A multilevel analysis based on gender role framework. | SSA Countries | 2016 - 2021 DHS | **3 Domains**  **Influencer:** Attitudes towards wife beating,  **Resource:** Occupation, having a mobile phone etc and  **Decision making:** On health care, major purchases & visiting family. |
| Tagang and Rwenge | 2023 | Women's autonomy and fertility in Chad. | Chad | Multiple Indicator Cluster Survey (MICS) & DHS | **1 Area:**  **Women's Autonomy:** Decision to visit family/relatives, health care, & large household purchases. |
| Sey-Sawo et al. | 2023 | Women's empowerment and nutritional status of children in the Gambia: further analysis of the 2020 Gambia demographic and health survey. | Gambia | DHS | **5 Indicators:**  Educational level,  E**mployment status:** Respondent currently working or not working,  **Decision making:** The person that decides on large household purchases, contraceptive use, and self-earnings,  **Age at first marriage and sex** and  **Acceptance of wife beating:** Beating justified if she went out without permission of husband, neglected the children, argued with husband, refused sex with husband, or burned the food. |
| Odwe et al. | 2023 | Women's empowerment and uptake of sulfadoxine-pyrimethamine for intermittent preventive treatment of malaria during pregnancy: results from a cross-sectional baseline survey in the Lake endemic region, Kenya. | Kenya | Revive IPTp-SP Baseline Survey | **4 Indicators:**  **Decision making power:** Person that decides regarding personal earnings, her healthcare, household purchases, and family visits,  **Control of productive assets**: land ownership, ownership of productive assets, having independent source of income, and making household purchase using their income,  **Education:** Education attainment and  **Employment status:** Working or not working. |
| Amoah et al. | 2023 | Influence of women empowerment on childhood (12-23 months) immunization coverage: Recent evidence from 17 sub-Saharan African countries. | 17 SSA Countries | DHS | **4 Indicators:**  **Labour force:** Working, not working,  **Acceptance of wife beating:** Neglect of a child, burning of food, arguing with husband, refuse sex, going out without permission,  **Decision making power:** Person who decides on respondent’s health care, house earning and household purchases and visiting family and  **Knowledge level:**  Listening to the radio, read newspaper, watch TV and educational level. |
| Zhou et al. | 2023 | Influences of women empowerment indices on demand for childcare services: Evidence from the Nigeria Demographic and Health Surveys. | Nigeria | DHS | **3 Domains**  **Influencer:** Attitudes towards wife beating,  **Resource:** Occupation, having a mobile phone etc and  **Decision making** Decision maker on respondent’s health care, major purchases & visiting family. |
| Sserwanja et al. | 2023 | Women empowerment indices and utilization of health facilities during childbirth: evidence from the 2019 Sierra Leone demographic health survey. | Sierra Leone | 2019 DHS | **3 Domains**  **Influencer:** Attitudes towards wife beating,  **Resource:** Occupation, having a mobile phone etc and  **Decision making** Decision maker on respondent’s health care, major purchases & visiting family. |
| Cardona et al. | 2024 | Measuring Women's Economic Empowerment in the Democratic Republic of Congo, Kenya, Nigeria, and Burkina Faso | 4 Low-&-middle income countries (DRC, Kenya, Nigeria, Burkina faso) | 2019 to 2021 Performance Monitoring for Action (PMA) | **2 Dimensions**  **Decision making:** Person who decide on large household purchases, daily purchase, medical purchases, clothes purchase and  **Financial autonomy:** Savings, financial information, financial goals. |
| Aboagye et al. | 2024 | Does women's empowerment and socio-economic status predict adequacy of antenatal care in sub-Saharan Africa? | 10 SSA Countries | 2018 - 2020 DHS | **2 Indicators**  **Decision making:** Decision maker on respondent’s health care, major purchases & visiting family and  **Justification for violence:** Burning food, arguing with him, going out without telling him, neglecting the children and refusing to have sexual intercourse with him. |
| Donkoh et al. | 2024 | Association between the survey-based women's empowerment index (SWPER) and intimate partner violence in sub-Saharan Africa. | 19 SSA Countries | DHS | **3 SWPER domains**  **Attitude to violence:** Beating justified wife neglects children, refuse to have sex, burn food,  **Decision making:** Decision on health care, & purchase of HH goods and  **Social independence:** Worked in last 12 months, age at cohabitation, age at first birth, & age difference between wife and husband. |
| Aboagye et al. | 2024 | Can women empowerment boost dietary diversity among children aged 6-23 months in sub-Saharan Africa? | 21 SSA Countries | DHS | **3 SWPER domains**  **Attitude to violence:** Beating justified wife neglects children, refuse to have sex, burn food,  **Decision making:** Decision on health care, & purchase of HH goods and  **Social independence:** Worked in last 12 months, age at cohabitation, age at first birth, & age difference between wife and husband. |
| Salihu et al. | 2024 | Spatial Heterogeneity and association between the survey-based Women's Empowerment Index (SWPER) and unmet need for birth spacing in sub-Saharan Africa. | SSA Countries | DHS | **3 SWPER domains**  **Attitude to violence:** Beating justified wife neglects children, refuse to have sex, burn food,  **Decision making:** Decision on health care, & purchase of HH goods and  **Social independence:** Worked in last 12 months, age at cohabitation, age at first birth, & age difference between wife and husband. |
| Dadzie et al. | 2024 | Association between women's empowerment and fertility preferences in Ghana. | Ghana | 2018 - 2022 DHS | **3 SWPER domains**  **Attitude to violence:** Beating justified wife neglects children, refuse to have sex, burn food,  **Decision making:** Decision on health care, & purchase of HH goods and  **Social independence:** Worked in last 12 months, age at cohabitation, age at first birth, & age difference between wife and husband. |
| Bamusi et al. | 2024 | Women's empowerment and its influence on the uptake of breast cancer screening in Tanzania: an analysis of 2022 Tanzania demographic health survey data. | Tanzania | 2022 DHS | **4 Variables**  **Property ownership:** Respondent own a house and land,  **Access to health care:** Distance to family, getting medical help,  **Decision making:** Decision maker on respondents’ health care, on large household purchases & on visits to family/friends and  **Attitude to violence:** Beating justified if wife goes out without telling husband, if she neglects the children, if she burns the food & if she argues with the husband. |
| Nibogore and Eryurt | 2024 | Women's Empowerment and Infant Mortality: Evidence from Rwanda. | Rwanda | 2005 to 2015 DHS | **3 Dimensions**  **Decision making:** Who usually makes decisions about health care for yourself, who usually makes decisions about making major household purchases? Who usually makes decisions about visits to your family, relatives and friends?  **Gender attitudes and beliefs:** If she goes out without telling him, if she neglects the children, if she refuses to have sex with him and if she burns the food and  **Human and Social Assets:** Women's education level, Women's occupation & Age at first marriage. |
| Eom et al. | 2024 | Women's empowerment and child anthropometric failures across 28 sub-Saharan African countries: A cross-level interaction by Gender Inequality Index. | 28 SSA Countries | DHS | **3 SWPER domains**  **Attitude to violence:** Beating justified wife neglects children, refuse to have sex, burn food,  **Decision making:** Decision on health care, & purchase of HH goods and  **Social independence:** Worked in last 12 months, age at cohabitation, age at first birth, & age difference between wife and husband. |
| Makongote et al. | 2024 | Women's Empowerment and Associated Factors in Kinshasa, Democratic Republic of Congo: A Secondary Data Analysis of the Performance Monitoring Assessment Survey. | DRC | Performance Monitoring Assessment Survey | **3 Domains**  **Safety and free from threat**s: Husband/partner influence,  **Control over sexuality:** Contraceptive use and  **Household decision making:** Large household purchases, daily purchases, health care, buying clothes, how partners earning will be used**.** |
| Achana et al. | 2024 | Women's autonomy, neonatal, infant and under-five mortality in the Upper East Region of Ghana. | Ghana | Cluster HH Survey | **1 Variable**  **Decision making autonomy:** Decision maker on daily purchases, large household purchases, can refuse sex, permission to health facility and visit family and friends. |
| Baye et al. | 2024 | Empowering women can improve child dietary diversity in Ethiopia. | Ethiopia | 2005-2016 DHS | **3 SWPER domains**  **Attitude to violence:** Beating justified wife neglects children, refuse to have sex, burn food,  **Decision making:** Decision on health care, & purchase of HH goods and  **Social independence:** Worked in last 12 months, age at cohabitation, age at first birth, & age difference between wife and husband. |
| Wassie et al. | 2024 | Women empowerment and childhood stunting: evidence from rural northwest Ethiopia. | Ethiopia | Primary data | **5 Dimensions**  **Decision Making:** Access to health care, household purchasing, freedom to visit relatives,  **Educational status:** No education, attend primary,  **Cash earning:** Respondent earn and not earn,  **Assets ownership:** Did not own a house, land and  **Membership in community groups:** Member and not member. |
| Hellwig et al. | 2024 | Association between women’s empowerment and demand for family planning satisfied among Christians and Muslims in multireligious African countries | 14 African countries | DHS | **3 SWPER domains**  **Attitude to violence:** Beating justified wife neglects children, refuse to have sex, burn food,  **Decision making:** Decision on health care, & purchase of HH goods and  **Social independence:** Worked in last 12 months, age at cohabitation, age at first birth, & age difference between wife and husband. |
| Salcedo et al. | 2025 | Measuring Multidimensional Women's Empowerment with Data From Partnered Women Across 45 Low- And Middle-Income Countries | 45 low middle income countries | DHS | **4 Dimensions**  **Health:** Sexual and reproductive health, control over health care,  **Material environment** Control over household purchases, control over earning,  **Social relationships:** Autonomy in social relationships, freedom of association and  **Physical integrity:** Acceptance of physical violence & Intimate partner violence. |
| Eom et al. | 2025 | Individual- and community-level women's empowerment and complete use of maternal healthcare services: A multilevel analysis of 34 sub-Saharan African countries | 34 SSA countries | 2011 to 2022 DHS | **3 SWPER domains**  **Attitude to violence:** Beating justified wife neglects children, refuse to have sex, burn food,  **Decision making:** Decision on health care, & purchase of HH goods and  **Social independence:** Worked in last 12 months, age at cohabitation, age at first birth, & age difference between wife and husband. |
| Yu et al. | 2025 | Women's Empowerment in Zimbabwe: Examining the Role of Educational Reform | Zimbabwe | 1999 to 2015 DHS | **3 Domains**  **Reproductive self determinations:** Age at first cohabitation, age at first birth,  **Household decision making:** Decision of family purchases, decision on contraceptive use,  **Domestic violence attitudes & experience:** Agreement on beating justified - by any reason or excuse, to have sex with partner, ever been through unwanted - sexual intercourse with partner, nonintercourse acts by partner. |
| Ayebeng et al. | 2025 | Cohabitation in sub-Saharan Africa: Does women empowerment matter? Insights from the demographic and health survey. | 13 Sub-Saharan Africa | DHS | **4 Domains**  **Labour Participation:** Working or not working  **Acceptance toward violence:** Justifying beating a wife, including going out without permission, neglecting children, arguing with the husband/partner, refusing sexual relations, and burning food,  **Decision making:** Healthcare, household earnings, purchases, and family visits and  **General knowledge level:** Education level and media consumption frequency. |
| Aboagye et al. | 2025 | Association between the Survey-based Women's Empowerment (SWPER) index and barriers to healthcare in sub-Saharan Africa. | 21 SSA Countries | 2015 - 2021 DHS | **3 SWPER domains**  **Attitude to violence:** Beating justified wife neglects children, refuse to have sex, burn food,  **Decision making:** Decision on health care, & purchase of HH goods and  **Social independence:** Worked in last 12 months, age at cohabitation, age at first birth, & age difference between wife and husband. |
| Abebe et al. | 2025 | The association of women's empowerment dimensions and antenatal care utilization in Ethiopia; facility based cross-sectional study. | Ethiopia | Primary data | **11 Dimensions**  Freedom of movement, Locus of control, Self-esteem, Self efficacy, Decision making, Justification of wife beating, Labour force participation, Information resource, Early marriage and Adolescence pregnancy. |
| Shibre et al. | 2025 | Level of and trends in women's empowerment inequalities in antenatal care services in Ethiopia: further analysis of the Ethiopia demographic and health surveys, 2000-16. | Ethiopia | 2000 -2016 DHS | **3 SWPER domains**  **Attitude to violence:** Beating justified wife neglects children, refuse to have sex, burn food,  **Decision making:** Decision on health care, & purchase of HH goods and  **Social independence:** Worked in last 12 months, age at cohabitation, age at first birth, & age difference between wife and husband. |
| Ahmed et al. | 2025 | Do empowerments influence experiencing intimate partner violence (IPV)? A multi-continental study of women across low and lower-middle-income countries. | 26 Middle income Countries | DHS | **5 Variables**  **Working status:** Working and not working,  **Employment status:** Seasonal, all-year, and occasional,  **Respondent's earning gap:** Partner no income, more than her, less than her, Same as her,  **Respondents’ autonomy:** Woman’s healthcare, significant household purchases, daily household needs, visits to family or relatives, daily food choices, and allocation of her partner’s earnings,  **Spending decisions:** Respondent alone, Respondent and partner, Partner alone, and Someone else. |
